# Serotonergic modulation of swallowing in a complete fly vagus nerve connectome

**DOI:** 10.1016/j.cub.2024.08.025

**Published:** 2024-09-12

**Authors:** Andreas Schoofs, Anton Miroschnikow, Philipp Schlegel, Ingo Zinke, Casey M. Schneider-Mizell, Albert Cardona, Michael J. Pankratz

**Affiliations:** 1 Department of Molecular Brain Physiology and Behavior, LIMES Institute, University of Bonn, Carl-Troll-Straße, Bonn 53115, Germany; 2 Department of Zoology, University of Cambridge, Downing Street, Cambridge CB2 TN1, UK; 3 MRC Laboratory of Molecular Biology, Cambridge Biomedical Campus, Francis Crick Avenue, Trumpington, Cambridge CB2 0QH, UK; 4 Allen Institute for Brain Science, Westlake Avenue North, Seattle, WA 98109, USA; 5 Janelia Research Campus, Howard Hughes Medical Institute, Helix Drive, Ashburn, VA 20147, USA; 6 Department of Physiology, Development and Neuroscience, University of Cambridge, Downing Place, Cambridge CB2 3EL, UK

## Abstract

How the body interacts with the brain to perform vital life functions, such as feeding, is a fundamental issue in physiology and neuroscience. Here, we use a whole-animal scanning transmission electron microscopy volume of *Drosophila* to map the neuronal circuits that connect the entire enteric nervous system to the brain via the insect vagus nerve at synaptic resolution. We identify a gut-brain feedback loop in which Piezo-expressing mechanosensory neurons in the esophagus convey food passage information to a cluster of six serotonergic neurons in the brain. Together with information on food value, these central serotonergic neurons enhance the activity of serotonin receptor 7-expressing motor neurons that drive swallowing. This elemental circuit architecture includes an axo-axonic synaptic connection from the glutamatergic motor neurons innervating the esophageal muscles onto the mechanosensory neurons that signal to the serotonergic neurons. Our analysis elucidates a neuromodulatory sensory-motor system in which ongoing motor activity is strengthened through serotonin upon completion of a biologically meaningful action, and it may represent an ancient form of motor learning.

## Graphical abstract

**Graphical abstract:**
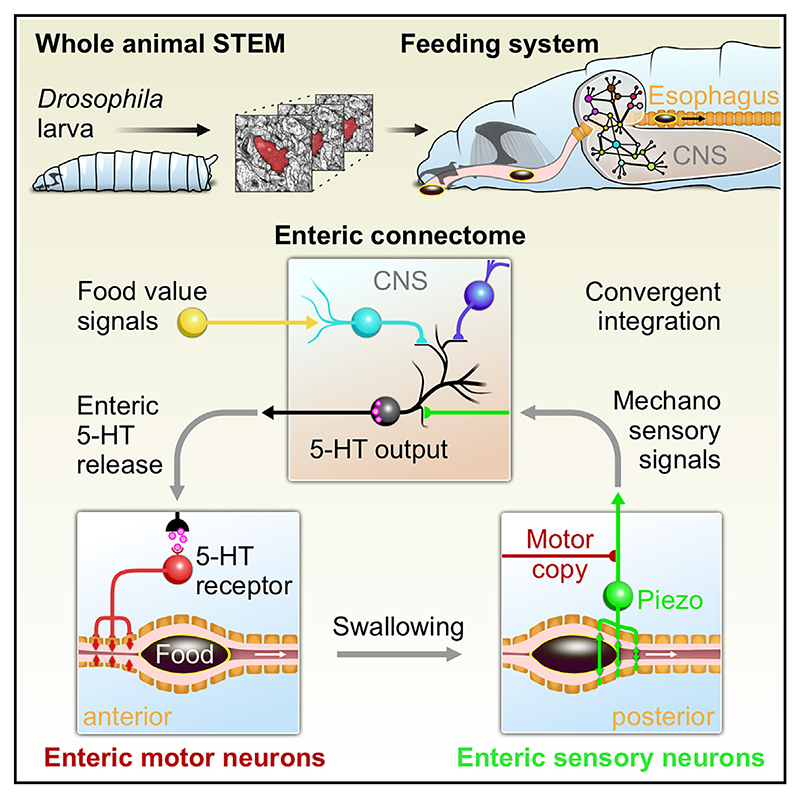

## Introduction

Feeding behavior entails interaction of the nervous system with environmental signals, as well as with physiological and metabolic signals provided by the internal organs. It can be seen as having different modules that form a chain of events, each of which requires a distinct set of motor programs.^1,2^ Different central pattern generators (CPGs) are thought to underlie particular motor programs for rhythmic feeding movements such as pharyngeal pumping, chewing, and swallowing.^3,4^ Critically, a feeding action should be reinforced when it successfully fulfills a biological need. This includes, for example, being able to distinguish swallowing movements that can occur through CPGs in the absence of sensory signals versus when real food is taken in, since the latter would have greater biological value for the organism.

The neuronal circuits underlying the various stages of food intake are currently being elucidated in different animals.^5–8^ CPGs for swallowing have been localized to the subesophageal zone (SEZ) in *Drosophila* and the brainstem in mammals,^9–11^ both of which are centers for processing sensory-motor information on taste- and feeding-related behaviors. Neurons involved in nutrient sensing and the neuronal pathways by which interoceptive signals are transmitted to the brain are being analyzed,^12–16^ including vagus nerve (VN) projections to the brainstem in mouse.^17–25^ In *Drosophila*, a strategy of identifying large numbers of specific cell types and of studying their function and projections to the central nervous system (CNS) has been employed.^26–28^ However, these approaches do not allow synaptic connections to be identified. In parallel, much progress has been made in characterizing the different cell types in the brain at single-cell level. For example, studies from various organisms and cellular contexts have shown that the neuromodulator serotonin has wide-ranging effects on feeding, gut motility, mood and motor learning,^29–34^ and the complexity of the serotonergic neurons in the mouse brain is being characterized.^30,35–37^ However, it is not known how the central serotonergic neurons are monosynaptically or polysynaptically connected to specific neurons of the periphery.

Connectomic analysis provides information on neuronal connectivity at synaptic resolution. In *Drosophila*, different datasets encompassing different parts of the CNS are being utilized.^38–44^ We have previously used the larval CNS volume to map the central circuits underlying motor and neuroendocrine control of feeding.^45–47^ A complete sensory and motor map has been reconstructed, including the topographical distribution of the sensory modalities from different body regions, with a distinct group of serotonergic neurons in the SEZ projecting to the enteric nervous system (ENS) and the gut.^47,48^ However, despite the level and comprehensiveness of the connectome analysis in the fly feeding system, there is a major gap that prevents the next level of understanding: one cannot match the synaptic partners between the periphery and the CNS at single-cell resolution, for neither the sensory nor the motor neurons. This knowledge would greatly advance understanding the brain map, since we can assign a biologically meaningful organ to which these projections belong. This would apply to mammalian systems as well, where synaptic mapping is being carried out for different brain regions in the mouse.^49^ A whole-animal electron microscopy (EM) volume, which includes the peripheral organs as well as the CNS, would fill this gap.

Here, in the first use of precisely such a whole-animal volume in *Drosophila*, we have reconstructed the complete ENS, assigning all connections from the peripheral feeding system to the brain at single-cell and synaptic level. These reaffirm the striking similarities between the circuit organization of mammalian and *Drosophila* VN,^48^ a term used previously to describe the meandering nerve that connects the CNS with the periphery in insects.^50^ Our results elucidate an elemental circuit for innate rhythmic behavior that utilizes serotonin to enhance a motor action in response to a successful, biologically valuable event, a process that we refer to as representing “action completion,” as a counterpart to action selection and initiation.^51^

## Results

### STEM reconstruction of the *Drosophila* VN and the ENS

**Figure 1:**
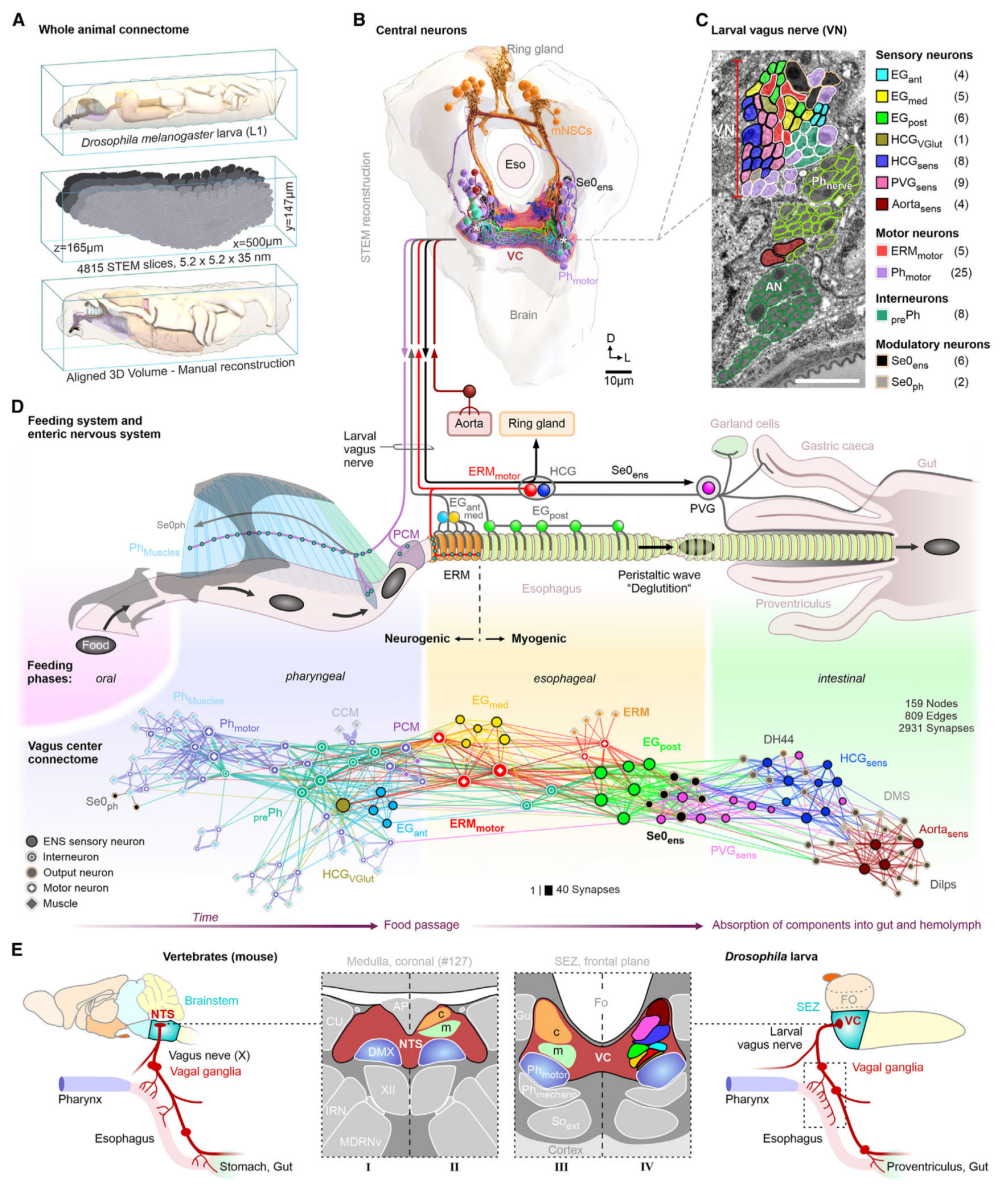
STEM reconstruction of the larval vagus nerve and the enteric nervous system (A) Whole first instar larva scanning transmission EM (STEM) volume consisting of 4,815 slices was used to completely reconstruct the feeding motor system and enteric nervous system (ENS). (B) Three-dimensional STEM reconstruction of the larval vagus center (VC) in the SEZ (frontal view), which is the primary synaptic integration site of the ENS onto different output neurons: motor neurons (Ph_motor_), modulatory neurons (Se0_ens_), and neurosecretory cells (mNSCs). (C) STEM cross-section of the compound nerve (referred to as “antennal nerve”), which includes the axon fiber bundle of the larval vagus nerve (VN). All neurons projecting through were identified and reconstructed as indicated by color coding. (D) Schematic drawing of the larval digestive tract and ENS (top) that is interconnected with larval VC in the brain by the larval VN (Schoofs et al.^48^; we refer here to the axonal pathway of CNS-ENS axis that projects through the antennal/recurrent nerve route as the VN). The ENS is composed of three vagal (enteric) ganglia. The EG includes 15 sensory neurons with dendrites covering the esophagus; the HCG includes 5 motor neurons innervating the ERM, 8 sensory neurons with local dendrites exposed to hemolymph but not associated with any tissue, and 1 VGlut-positive, putative interneuron; and the PVG includes 9 sensory neurons associated with the midgut and metabolic organs, e.g., the gastric caeca and the nephrocyte-like garland cells.^53^ In addition, 9 neuromodulatory neurons in the PVG establish a connection between midgut and ring gland. Force-directed atlas of the VC connectome in the CNS based on synaptic connectivity correlates with the four coordinated phases of food intake behavior (bottom). (E) Comparison of the central representation of vagal afferents in mouse (I, II) and *Drosophila* larva (III, IV). In both species, vagal mechanosensory (m) and chemosensory (c) inputs project to adjacent but distinct sub-areas in brainstem (mouse) and SEZ (*Drosophila*). Using STEM reconstruction, the projection fields of these afferents could be ascribed to functional groups of individual enteric sensory neurons in *Drosophila*. Abbreviations: AP, area postrema; Aorta_sens_, sensory neurons of the aorta; CU, cuneate nucleus; DH44, diuretic hormone 44; Dilps, *Drosophila* insulin-like peptides; DMS, drosomyosuppresin; DMX, dorsal motor nucleus of the vagus nerve; EG_ant/med/post_, esophageal ganglion (anterior, medial, and posterior); ERM, esophageal ring musculature; ERM_motor_, esophageal ring musculature motor neurons; Fo, foramen; Gu, gustatory afference; HCG_sens/VGlut_, hypocerebral ganglion (sensory neurons, VGlut-positive neuron); IRN, intermediate reticular nucleus; MDRNv, ventral medullary reticular nucleus; NTS, nucleus of the solitary tract; PCM, pharyngeal constrictor musculature; Ph_mechano_, pharyngeal mechanosensory afference; Ph_Muscles_, pharyngeal musculature; Ph_motor_, pharyngeal motor neurons; _Pre_Ph, pharyngeal premotor neurons; PVG_sens_, proventricular ganglion (sensory neurons); Se0_ens_, enteric Se0 neurons; Se0_ph_, pharyngeal Se0 neurons; So_ext_, external somatosensory afference; SEZ, subesophageal zone; VC, vagus center; XII, hypoglossal nucleus. See also Figure S1.

A whole-animal scanning transmission EM (STEM) dataset of a *Drosophila* larva^52^ (Figure 1A) was used to fully reconstruct all neurons and target organs of a nerve that connects the ENS with the brain (Figures 1B and 1C). We refer to this nerve as the VN, based on previous usage and analysis.^48,50^ The larval VN splits into two major branches after it exits the brain. One projects anteriorly onto the pharyngeal muscles, while the other projects posteriorly toward the midgut; these interconnect the distinct ganglia of the ENS (esophageal, hypocerebral, and proventricular ganglia [PVG]) and the major endocrine organ (ring gland). Serotonergic neurons of the VN also have two clusters, one that projects to the pharynx (Se0_ph_) and the other that projects to the midgut (Se0_ens_).

The sensory, motor, and neuromodulatory neurons of the VN will be discussed individually in subsequent sections. As an overview, the sensory axons with their presynaptic sites project to a special region of the SEZ, which we term the vagus center (VC) (Figure 1B). The somata of the motor neurons that innervate the pharyngeal muscles are localized in the SEZ that borders the VC, whereas motor neurons that innervate the esophageal muscles are localized peripherally in the hypocerebral ganglion (HCG). We also uncovered a novel sensory organ (“aorta sensory neurons” [Aorta_sens_]) that innervates the aorta and sends projections to the SEZ and the neurosecretory cells in the brain (Figures 1B and S1A). We also reconstructed all peripheral organs comprising the feeding apparatus that underlie the oral, the pharyngeal, and the esophageal phases of deglutition,^54^ similar to those of mammals.^55^ Next, all direct connections between the enteric sensory neurons and the swallowing-related output neurons were determined. These revealed that the muscle system drives swallowing transitions from neurogenic to myogenic control along the esophagus (Figure 1D). Finally, force-directed graph mapping based on monosynaptic connectivity showed a concatenated series of circuit modules that is aligned along the foregut axis, reflecting the temporal flow of food passage corresponding to the sequential phases of food intake (Figures 1D and S1B–S1D).

Together with earlier synaptic mapping of the larval feeding system,^45–47^ the current whole-animal analysis enables a comparative view with the organization of the mammalian vagal sensory inputs to the brain (Figure 1E). Classical anatomical work has defined topographically separated vagal sensory projections onto the nucleus of the solitary tract (NTS), which could be further subdivided into distinct target regions based on sensory modality and peripheral origin.^23^ These include the adjacent but clearly separated vagal mechanosensory and chemosensory projections from the gastrointestinal tract via the nodose ganglion. For both *Drosophila* and mouse, the motor neurons occupy a distinct region that abuts the sensory projection target (Figures 1E, S1B, and S1C). The basic organization of the modality and organ-specific sensory projections to the brain, re-inforced here at synaptic and single-cell resolution, illustrates the similarities between the vertebrate and *Drosophila* VNs.

### Mapping serotonergic active zones in the ENS

**Figure 2:**
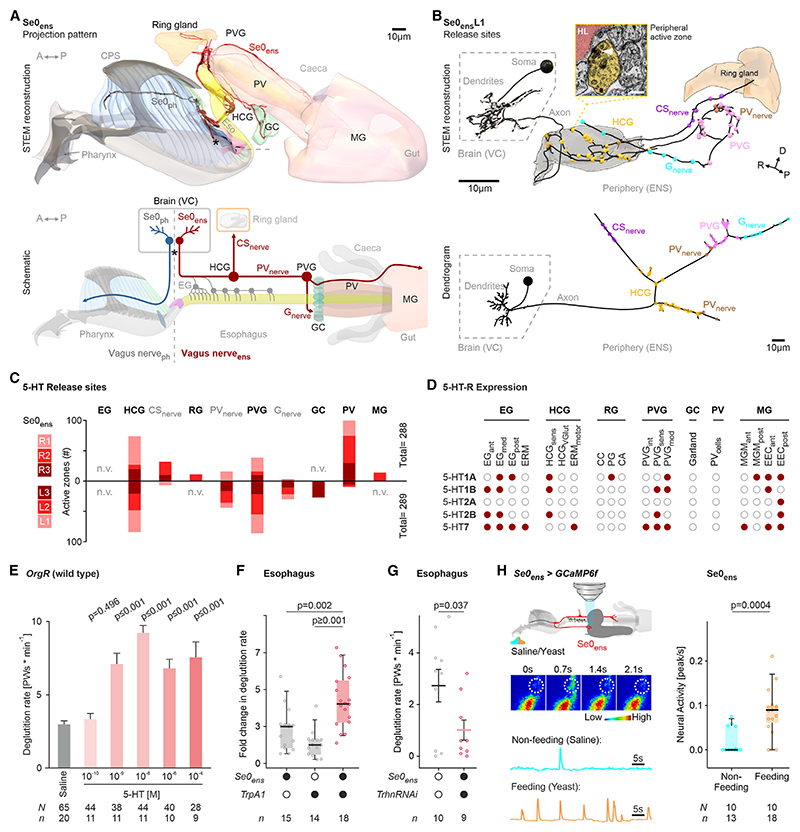
Serotonergic output neurons of the enteric nervous system (A) Three-dimensional image of the STEM reconstruction (top) and schematic illustration (bottom) of digestive tract and ENS, marking the targets of Se0_ens_ (red) and Se0_ph_ (blue) peripheral active zones. (B) Three-dimensional image (top) and dendrogram (bottom) of Se0_ens_ neuron (L1); colored circles indicate peripheral active zones. Inserted EM image shows a typical peripheral active zone (presynaptic site without postsynaptic partners) of a Se0_ens_ neuron (scale bar, 0.2 μm). (C) Spatial distribution of serotonergic peripheral active zones of all Se0_ens_ neurons onto digestive tract, ENS, and endocrine organs. (D) Serotonin receptor expression analysis of ENS (Figure S3 and Tables S1 and S2). (E) Dose-dependent increase of PWs per minute after bath application of serotonin (data show mean and ± SE). Performed significance test: Mann-Whitney rank-sum test. (F) Activating Se0_ens_ neurons accelerates PWs per minute, compared with controls. Performed significance test: Mann-Whitney rank-sum test. (G) Blocking serotonin synthesis in Se0_ens_ neurons by RNAi against tryptophan hydroxylase (Trhn) reduces PWs per min (data show mean and ±SE). Performed significance test: Mann-Whitney rank-sum test. (H) Left, top: calcium-imaging setup of Se0_ens_ neurons in non-feeding and feeding state. Left, middle: representative images of Se0_ens_ neuron recording; dashed circle indicates the region of interest for the analysis. Left, bottom: calcium recording of Se0_ens_ neuron in non-feeding (cyan trace) and feeding (orange trace) states. Right: neural activity of Se0_ens_ neurons in non-feeding and feeding animals. Note the significant increase in neural activity of Se0_ens_ neurons in the feeding state. Performed significance test: Mann-Whitney rank-sum test. Abbreviations: CA, corpora allata; CC, corpora cardiaca; EEC_ant/post_, anterior/posterior entero-endocrine cells; EG_ant/med/post_, esophageal ganglion (anterior, medial, and posterior); ENS, enteric nervous system; ERM, esophageal ring musculature; ERM_motor_, ERM motor neuron; GC, garland cells; G_nerve_, garland nerve; HCG_VGlut/sens_, hypocerebral ganglion (VGlut, sensory); MG, midgut; MGM_ant/post_, midgut musculature (anterior, posterior); CS_nerve_, nervus cardio stomatogastricus; PG, prothoracic gland; PV, proventriculus; PV_nerve_, proventricular nerve; PVG_int/mod/sens_, proventricular ganglion (intrinsic, modulatory, and sensory); PW, peristaltic wave; RG, ring gland; Se0_ens_, enteric Se0 neurons; Se0_ph_, pharyngeal Se0 neurons. See also Figures S2 and S3 and Tables S1–S3.

As a seed for functionally analyzing neuronal circuits underlying brain-gut interactions via the VN, we focused on the enteric Se0 neurons (Se0_ens_) that project posteriorly to the enteric ganglia, the ring gland, and the midgut^48^ (Figures 2A and S2A). Interestingly, Se0_ens_ have peripheral presynaptic-like sites that lack distinct postsynaptic sites, which we term “peripheral active zones” (Figures 2B and S3A–S3D). These may represent an intermediate structure between the classical presynaptic and postsynaptic pairs and the neuropeptide releasing cells that lack presynaptic structures.^45^ We annotated each individual peripheral active zone of the Se0_ens_ neurons and assigned a position tag relative to the ENS or its innervation targets (Figures 2B and 2C). This showed that the primary targets of the Se0_ens_ neurons are the HCG and PVG.

Expression analysis of all five serotonin receptors, using knockin T2A-Gal4 driver lines,^56^ showed that serotonin receptors are widely distributed throughout the ENS and gut-associated peripheral organs (Figures 2D and S3E–S3J; Tables S1 and S2); different subtypes are expressed in specific subsets in the sensory and motor neurons, as well as in the muscles and endocrine cells. Addition of serotonin, as well as thermogenic activation of Se0_ens_ neurons in *ex vivo* experiments, increased deglutition (measured as esophageal peristalsis per minute) (Figures 2E and 2F), while targeted inactivation of serotonin synthesis in Se0_ens_ neurons by knocking down *Trhn* decelerated deglutition (Figure 2G). GCaMP recordings of Se0_ens_ under fed and non-fed conditions showed that active feeding increased the neuronal activity of Se0_ens_ (Figure 2H). Se0_ens_ also increase activity in response to attractive nutrients and decrease activity to bitter compounds (Figures S2B and S2C). Taken together, these results indicate that Se0_ens_ are involved in modulating deglutition, and their activity is regulated by nutrient signals and feeding state.

### Peripheral enteric motor neurons controlling deglutition project to esophageal muscles and CNS

**Figure 3:**
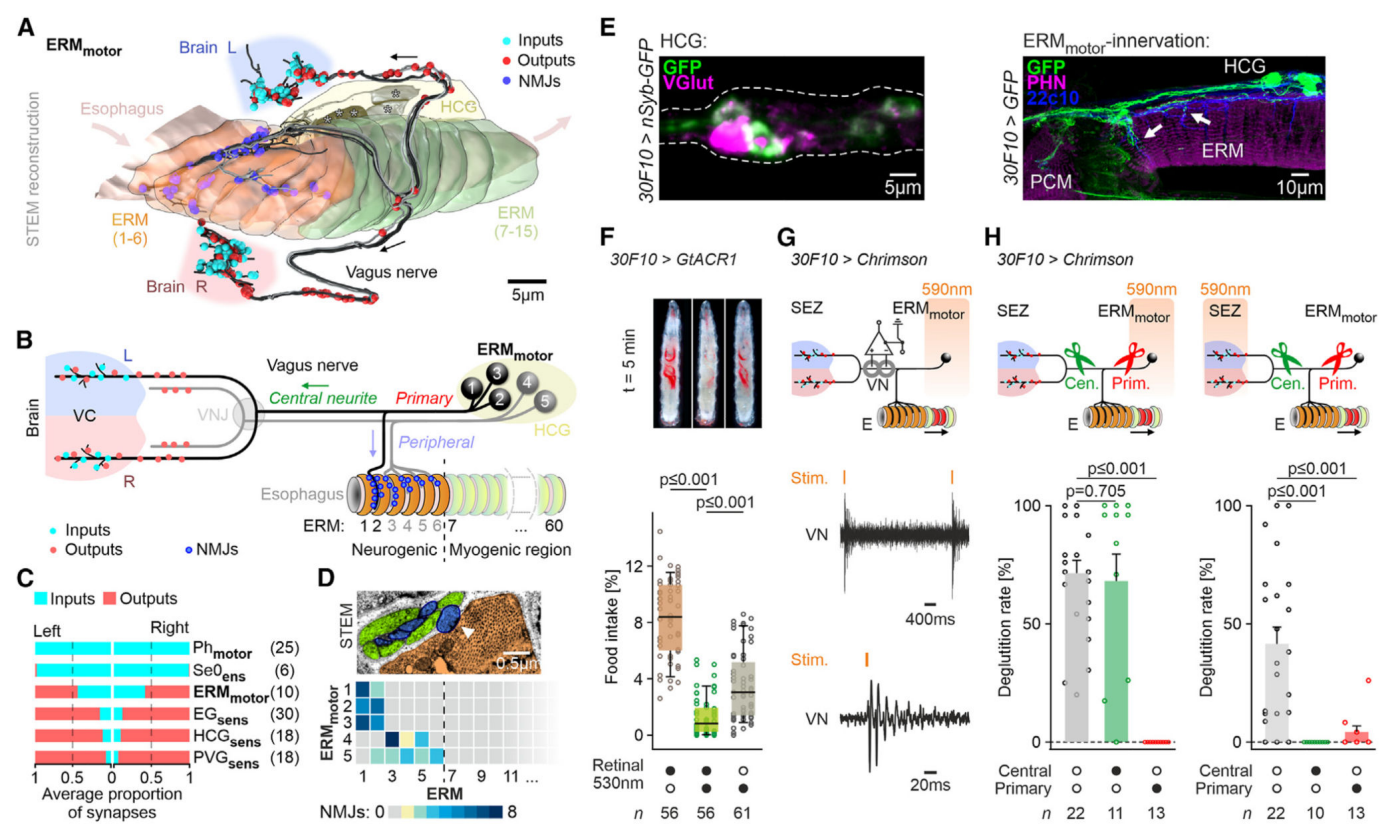
Motor neurons innervating the esophagus and their connection to the CNS (A) Three-dimensional image of the reconstructed ERM motor neurons (ERM_motor_) showing the peripheral NMJs (purple) and central synaptic inputs (cyan)/ outputs (red) with the esophagus, including the ERMs. Somata of ERM_motor_ in the HCG are marked with asterisks. (B) Schematic of ERM_motor_. Primary neurite extends out from the cell body located in the HCG and bifurcates into a peripheral neurite innervating the ERM (at the NMJs) and into a central neurite connecting the ERM_motor_ via the vagus nerve to the brain (synaptic inputs and outputs). Note that the esophagus is divided into a neurogenic region (ERM1–6) and a myogenic region (ERM7–60). (C) Relative degree of input to output synapses in the CNS for sensory (input) neurons and modulatory/motor (output) neurons (number in parentheses stands for the total number of central neurites per neuron type). Note the hybrid input to output proportion of ERM_motor_. (D) Top: STEM slice of a motor axon (blue) with NMJ (arrowhead) on a striated ERM (orange). Bottom: heatmap shows the number of NMJs per ERM for each ERM_motor_. NMJs are restricted to ERM1–6. Note the decreasing innervation of ERMs (number of NMJs per ERM) along the anterior-posterior axis of the esophagus. (E) Left: staining of *30F10* > *nSyb-GFP* shows four cell bodies in the HCG that are co-localized with the VGlut antibody signal. One VGlut-positive neuron shows no co-localization, which is the VGlut-positive HCG neuron (HCG_VGlut_). Right: GFP-expression of *30F10-Gal4* covers ERM_motor_ in HCG showing the peripheral neurites targeting the ERMs (arrows). (F) Inhibition of ERM_motor_ by GtACR1 suppresses food swallowing. Representative images (top) and boxplot (bottom) of larval food intake with/without ERM_motor_ inhibition. Performed significance test: Mann-Whitney rank-sum test. (G) Activation of ERM_motor_ in ENS by Chrimson elicits afferent spikes detectable in VN recordings. (H) Left: ablating the central neurite (Cen) and activating ERM_motor_ in ENS induced peristalsis, whereas ablation of primary neurite (Prim) abolished peristalsis. Right: ablating either the central neurite (Cen) or primary neurite (Prim) and activating the SEZ, abolished peristalsis (data show mean and ±SE). Performed significance test: Mann-Whitney rank-sum test. Abbreviations: Dist, distal ablation (central neurite); E, esophagus; EG_sens_, esophageal ganglia (sensory neurons); ERM, esophageal ring musculature; ERM_motor_, ERM motor neuron; HCG_sens_, hypocerebral ganglia (sensory neurons); L, left; NMJ, neuromuscular junction; Ph_motor_, pharyngeal motor neurons; PCM, pharyngeal constrictor musculature; PHN, phalloidin; Prox, proximal ablation (primary neurite); PVG_sens_, proventricular ganglia (sensory neurons); R, right; SEZ, subesophageal zone; Se0_ens_, enteric Se0 neurons; Stim, stimulus; VC, vagus center; VN, vagus nerve; VNJ, vagus nerve junction. See also Figure S4 and Table S3.

We next identified the motor neurons that innervate the esophageal ring musculature (ERM) to trigger deglutition (Figure 3A). These pseudounipolar motor neurons (ERM_motor_) have a striking morphology and projection pattern: from their somata in the HCG, the primary neurite divides into a peripheral neurite innervating the ERM and a central neurite projecting bilaterally to the brain (Figure 3B). The central neurites in the SEZ have nearly equal amounts of input and output synapses, placing them as intermediates between sensory and motor neurons (Figure 3C). Notably, only the anterior six ERMs are innervated, with the number of neuromuscular junctions (NMJs) per ERM decreasing posteriorly (Figures 3B and 3D). This subdivides deglutition into a “voluntary” (neurogenic) region and an “involuntary” (myogenic) region.

A Janelia Gal4 line (*30F10-Gal4*) drives expression in the glutamatergic ERM_motor_ (Figures 3E and S4A), and optogenetic inhibition of the ERM_motor_ using GtACR1 led to a significant reduction of food intake (Figure 3F), showing their requirement in feeding behavior. To further investigate the functional role of the ERM_motor_, we first activated these neurons optogenetically at their somata, while monitoring deglutition and recording extracellularly from the larval VN (Figure 3G). This elicited an afferent signal in the VN, indicating a functional connection to the CNS (Figure 3G). To see whether the connection to the CNS was required for deglutition, we lesioned the central neurite and activated the ERM_motor_ at the soma (Figure 3H, left). Deglutition rate was not altered, indicating that CNS projection is not required for activation of ERM_motor_. By contrast, lesioning the primary neurite abolished deglutition (Figure 3H, left). When the ERM_motor_ was specifically activated in the SEZ (Figure 3H, right), lesioning either the central neurite or the primary neurite abolished deglutition. Taken together, these results showed that ERM_motor_ control esophageal peristalsis and send afferent signals to the brain.

### Enteric motor neurons are modulated by serotonin

**Figure 4:**
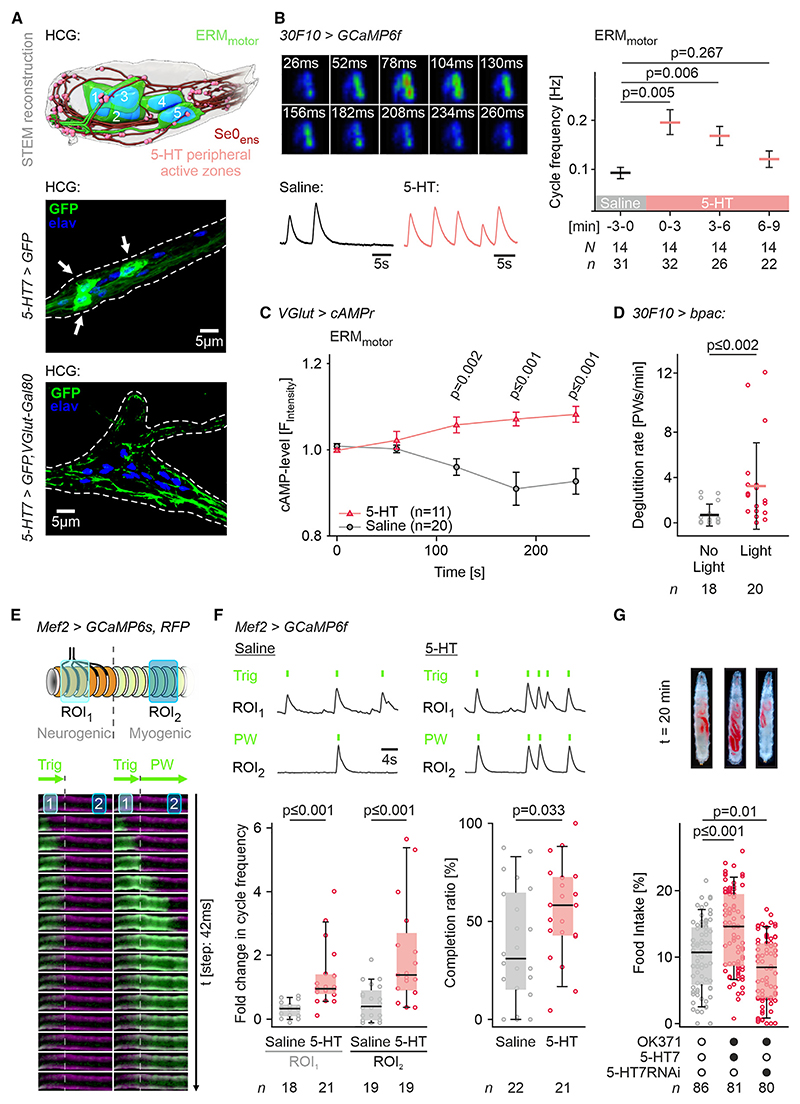
Serotonin signaling in the ERM motor system (A) Top: three-dimensional drawing of five ERM_motor_ and the projections/peripheral active zones of Se0_ens_ in the HCG. Middle: expression of 5-HT7 showing three neurons in HCG. Bottom: expression of 5-HT7 in presence of the Gal4-repressor Gal80 driven by *VGlut* promoter shows no neurons in HCG. Indicates that three ERM_motor_ neurons express 5-HT7. (B) Left: calcium imaging of ERM_motor_; representative data showing the effect of adding serotonin on ERM_motor_. Right: analysis shows increased neuronal activity in ERM_motor_ after adding serotonin (data show mean and ± SE). Performed significance test: Mann-Whitney rank-sum test. (C) cAMP reporter (cAMPr) showed, after serotonin treatment, an increased cAMP level in ERM_motor_ (data show mean and ± SE). Performed significance test: Mann-Whitney rank-sum test. (D) Optogenetic increase of cAMP level in ERM_motor_ by *UAS-bpac* accelerated deglutition (data show mean and ± SE). Performed significance test: Mann-Whitney rank-sum test. (E) Calcium imaging of ERMs reveals two distinct active muscle zones: neurogenic zone (ROI_1_) and myogenic zone (ROI_2_). Based on the observation that no contraction wave occurred in the myogenic region without onset in the neurogenic region, we defined muscle activity in ROI_1_ as a trigger (Trig) and ROI_2_ as a complete peristaltic wave (PW). (F) Top: calcium recording of ROI_1_ (neurogenic) and ROI_2_ (myogenic), showing the effect of serotonin on ERM activity. Green lines mark muscle activity in ROI_1_ (Trig) and ROI_2_ (PW). Bottom: analysis shows an increase in ERM cycle frequency at ROI_1_ and ROI_2_ after application of serotonin (left). Analysis shows an increase in completion rate after application of serotonin (right). Performed significance test: Mann-Whitney rank-sum test. (G) 5-HT7 overexpression in motor neurons by *OK371-Gal4* increased food intake, whereas the knockdown of 5-HT7 decreased food intake. Performed significance test: Mann-Whitney rank-sum test. Abbreviations: cAMP, cyclic adenosine monophosphate; ERM_motor_, ERM motor neuron; HCG, hypocerebral ganglion; PW, peristaltic wave; ROI, region of interest; Se0_ens_, enteric Se0 neurons; Trig, Trigger. See also Figure S4 and Table S3.

ERM_motor_ are surrounded by the peripheral active zones of the Se0_ens_ in the HCG (Figure 4A, top). We show that the 5-HT7 receptor is expressed in the ERM_motor_ by co-expressing VGlut-Gal80, which can repress 5-HT7-Gal4 expression, in glutamatergic neurons (Figure 4A, middle and bottom). Addition of serotonin increases the neural activity of the ERM_motor_ (Figure 4B), with the highest increase in the first 3 min. As the excitatory 5-HT7 is the only serotonin receptor that increases the intracellular cyclic adenosine monophosphate (cAMP) level by activating adenylate cyclase,^57^ we applied serotonin onto semi-intact larva expressing a cAMP sensor (cAMPr) in ERM_motor_ and observed an increase in intracellular cAMP level in the ERM_motor_ (Figure 4C). Similar results were obtained using a different cAMP sensor (Epac-camps; Figure S4B). Optogenetically increasing the cellular cAMP level in the ERM_motor_ by using the photoactivatable adenylate cyclase, bPAC^58^ increased deglutition (Figure 4D). These results in combination strengthen the view that ERM_motor_ are modulated by serotonin.

Next, we determined how serotonin modulates ERM activity by monitoring the contraction waves of the ERMs using GCaMP6f. Strikingly, two distinct patterns of muscle activity were observed: one being restricted to the neurogenic region (Figure 4E, left) and the other, which includes both the neurogenic and the myogenic regions, reflecting a completion of peristaltic wave (Figure 4E, right). Contraction in the neurogenic region (trigger signal) often occurs without progressing to the myogenic region. However, we have never observed contraction waves in the myogenic region without prior trigger signal in the neurogenic region. Adding serotonin resulted in increased activity in both the neurogenic and myogenic regions. Importantly, serotonin increased the occurrence of completed contraction waves relative to the triggered ones (“completion ratio”) (Figure 4F).

To verify the role of serotonin *in vivo*, we genetically manipulated the expression of 5-HT7 in glutamatergic ERM_motor_. Over-expression of 5-HT7 in all glutamatergic neurons (via *OK371* driver) increased food intake; conversely, a knockdown of 5-HT7 expression decreased food intake (Figure 4G). Taken together, these data indicate that serotonin signaling in the ERM_motor_ via the receptor 5-HT7 modulates deglutition and food intake.

### Different classes of esophageal sensory neurons have distinct functions in swallowing

**Figure 5:**
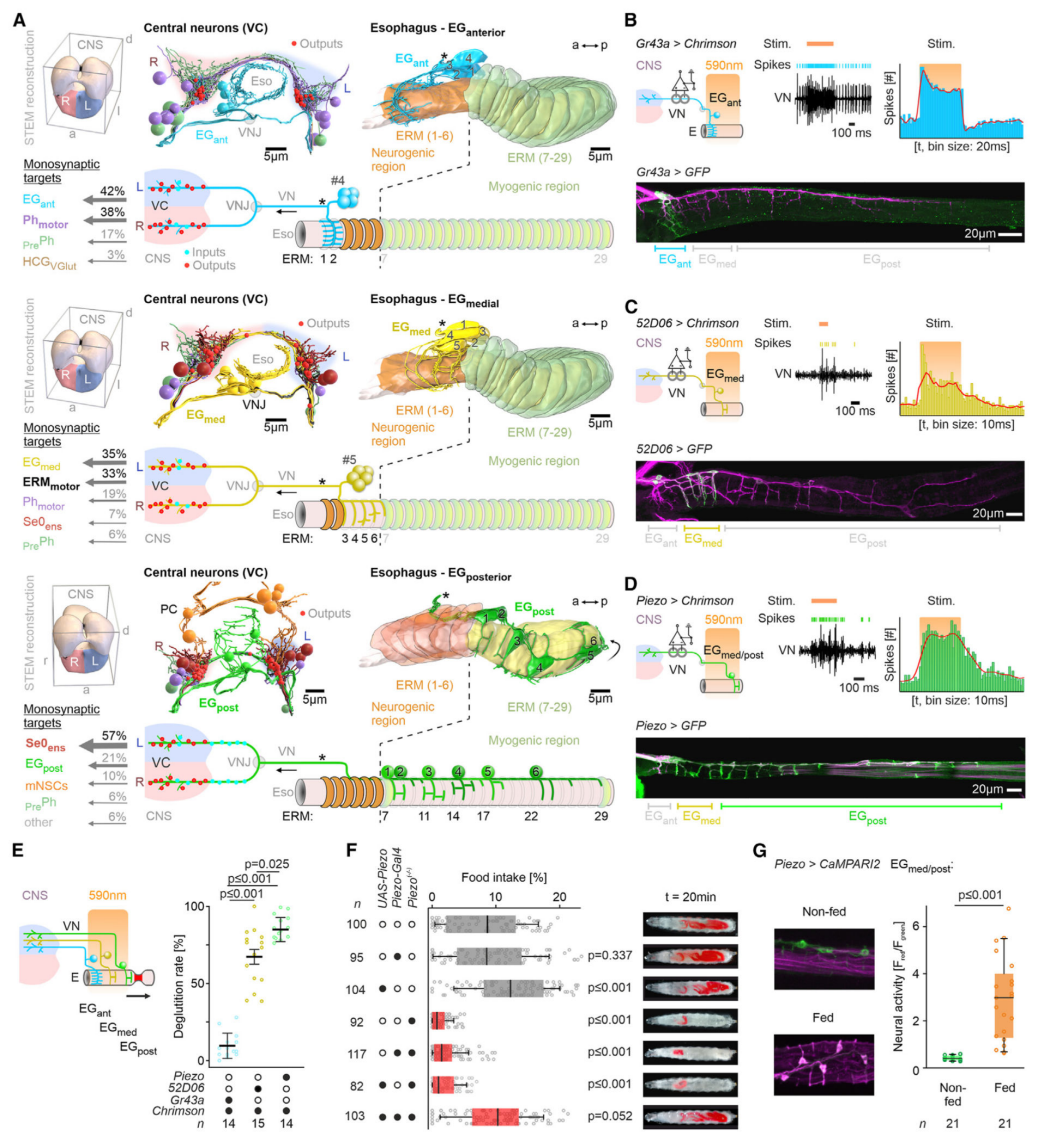
Different clusters of sensory neurons in the esophagus have distinct roles in swallowing (A) STEM reconstruction and schematic illustration of EG_ant_ (top, blue), EG_med_ (middle, yellow), and EG_post_ (bottom, green), showing the receptive fields on the esophagus and the monosynaptic (direct) targets in the VC. Percentage shows the fraction of total outgoing monosynaptic budget. EG_ant_ primarily targets Ph_motor_ in addition to having strong intrasensory connections. Dendritic field of EG_ant_ covers ERM1/2 in the neurogenic region. EG_med_ mainly targets ERM_motor_, plus strong intrasynaptic connections. Dendritic field of EG_med_ covers ERM3–6 in the neurogenic region. EG_post_ shows primary synaptic outputs to Se0_ens,_ with lower degree of intrasynaptic connections. Dendritic field of EG_post_ covers the myogenic region from ERM7–29. (B) Activation of EG_ant_ by *Gr43a* > *Chrimson* induces afferent series of spikes recorded in the VN (top). Chemoreceptor Gr43a is expressed in EG_ant_ (bottom). (C) Activation of EG_med_ by *52D06* > *Chrimson* induces afferent spikes recorded in the VN (top). *52D06-Gal4* drives expression in the EG_med_ (bottom). (D) Activation of EG_post_ by *Piezo* > *Chrimson* induces afferent spikes recorded in the VN (top). Mechanoreceptor Piezo is expressed in EG_med_ and EG_post_ (bottom). (E) Activation of EG_med_ and EG_med/post_ elicits peristalsis, whereas activation of EG_ant_ does not. Data are mean ± SD. Performed significance test: Mann-Whitney rank-sum test. (F) *Piezo*^*(*−*/*−*)*^ larvae showed reduced food intake that is rescued by expression of Piezo via Gal4/UAS-system in *Piezo*^*(*−*/*−*)*^ background. Gal4-driver and UAS-effector were tested in a wild-type and *Piezo*^*(*−*/*−*)*^ background. Only the UAS-effector line in the wild-type background showed increased food intake, compared with the wild-type line, presumably caused by the genetic insertion. Data are shown as boxplots. Performed significance test: one-way ANOVA test. (G) CaMPARI experiments of EG_med/post_ using *Piezo*^*(KI)*^*-Gal4* showed an increase in neural activity during feeding, suggesting that mechanosensory EG neurons are able to monitor food passage through the esophagus. Performed significance test: Mann-Whitney rank-sum test. Abbreviations: EG_ant/med/post_, esophageal ganglion (anterior, medial, and posterior); ERM, esophageal ring musculature; ERM_motor_, ERM motor neurons; HCG_VGlut_, hypocerebral ganglion (VGlut-positive neuron); mNSC, medial neurosecretory cell; Ph_motor_, pharyngeal motor neurons; _Pre_Ph, pharyngeal premotor neurons; Se0_ens_, enteric Se0 neurons; Stim., stimulus; VC, vagus center; VN, vagus nerve; VNJ, vagus nerve junction. See also Figure S5 and Table S3.

We next identified all sensory neurons with dendritic contact to the esophagus, as well as their monosynaptic targets (Figure 5A). Clustering by neuronal morphology and synaptic connectivity revealed three different clusters of neurons in the esophageal ganglia (EG anterior, medial, and posterior) along the anterior-posterior axis: EG_ant_, EG_med_, and EG_post_ (Figures 5A, S5A, and S5B).

Several salient features with functional implications can be discerned. First, the EG_ant_ and EG_med_ neurons innervate distinct domains in the neurogenic region: EG_ant_ innervates ring muscles 1 and 2, while EG_med_ innervates 3 to 6 (from a total of 60 ring muscles). In striking contrast, the EG_post_ neurons innervate only the myogenic region (tandemly arrayed across muscles 7 to 29). Second, the monosynaptic targets of the three clusters are very different. EG_ant_ neurons are highly connected to pharyngeal motor neurons (Ph_motor_) and premotor neurons (_Pre_Ph). The EG_med_ neurons, on the other hand, have strong monosynaptic contacts to ERM_motor_. The most distinct are the EG_post_ neurons, which have a strong monosynaptic connection to Se0_ens_ but essentially none to the pharyngeal or esophageal motor neurons. Third, the distinct esophageal clusters show strong reciprocal intracluster synaptic connections, suggesting that neural response of one neuron conditions the activity of all neurons within a cluster, likely resulting in an amplification or synchronization of sensory inputs to their targets.

We then identified Gal4 lines that show expression patterns in different subsets of EG neurons (Figures 5B–5D). The chemoreceptor Gr43a is expressed in EG_ant_, the Janelia 52D06 line is expressed in EG_med_, while the mechanoreceptor Piezo is expressed in EG_med_ and EG_post_. Optogenetic activation of each of the three lines elicited spikes in the VN, indicating that an afferent signal is transmitted to the CNS (Figures 5B–5D). The effect on deglutition, however, was quite different. Activation of EG_ant_ (via *Gr43a-Gal4*) did not induce deglutition (Figure 5E). Considering its predominant monosynaptic connectivity to the pharyngeal motor system (Ph_motor_ and _Pre_Ph), this suggests that EG_ant_ is involved in the coordination of swallowing between the pharynx and the esophagus, but not in deglutition per se. Activation of EG_med_ (via *52D06-Gal4*; Figure S5C) strongly induced deglutition (Figure 5E); together with the fact that EG_med_ has direct synaptic connections to the ERM_motor_, this indicates that the EG_med_ neurons are involved in the initiation of deglutition. Activation of both EG_med_ and EG_post_ neurons (EG_med/post_ via *Piezo-Gal4*) had only induced deglutition slightly more than activation of EG_med_ alone (Figure 5E); this indicates that EG_post_ likely has an indirect role in deglutition by acting through Se0_ens_, which then acts on the ERM_motor_. Since EG_med_ and EG_post_ express the Piezo mechanoreceptor, we asked whether *Piezo* gene activity is required for food intake. Indeed, *Piezo*^*(*−*/*−*)*^ animals had significantly decreased food intake, which could be rescued by reintroduction of a *Piezo* transgene (Figure 5F). Functional imaging analysis using the calcium integrator CaMPARI further showed that the *Piezo*-expressing EG neurons are activated in fed condition (Figures 5G and S5D), showing the importance of mechanosensory input for deglutition and feeding *in vivo*.

### Mapping all monosynaptic connections between sensory input and effector output cells of the ENS

**Figure 6:**
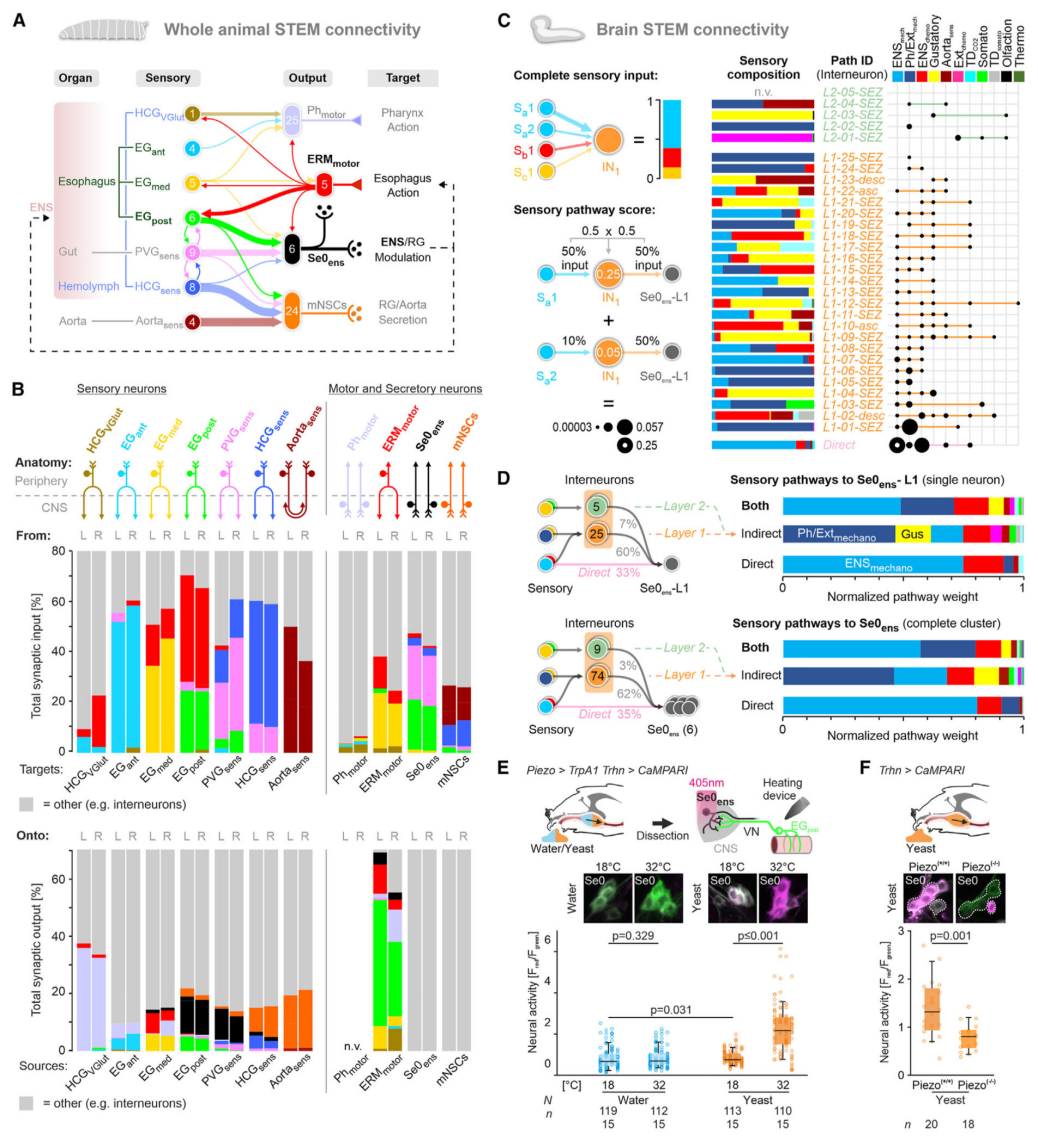
Monosynaptic sensory-output connectivity of the ENS and interneuronal pathways onto the Se0_ens_ neurons (A) Illustration of all monosynaptic (direct) connections between the ENS including Aorta_sens_ neurons and the four primary feeding-related neuronal output systems based on the whole-animal STEM volume. (B) Top: basic structure of the listed neuron cluster is represented as an arrow diagram. Note that all enteric neurons bilaterally project to CNS, except Aorta_sens_. Bottom: colored bar graphs show the total synaptic input and output for the left/right brain hemisphere of the sensory, motor, and secretory neuron clusters in the vagus connectome. (C) Analysis of all indirect sensory pathways onto single Se0_ens_-L1 taken from the brain connectome.^47^ Top, left: complete sensory inputs of an individual interneuron (IN_1_) can be grouped according to their sensory modality (defined here as sensory composition). Bottom, left: sensory pathway score is the product of the normalized input fraction of a sensory neuron onto an interneuron (IN_1_) and the normalized input fraction of IN_1_ onto the target, Se0_ens_-L1. Scores of IN_1_ are summed for each sensory modality. Right: grid matrix shows the sensory pathway scores for direct and indirect sensory pathways onto the Se0_ens_-L1 neuron. For the indirect sensory pathways onto Se0_ens_-L1, the sensory composition of all interneurons connected to Se0_ens_-L1 is represented as a bar plot. Integrated sensory modalities onto Se0_ens_-L1 are shown as dots (size represents pathway score) and arranged according to their total path score. (D) Left: schematic of all direct and indirect sensory pathways onto Se0_ens_-L1 (top) and all Se0_ens_ (bottom) taken from the brain connectome. Numbers within the circles represent the number of neurons in the interneuron layer (L1/L2). Percentage values represent the synaptic input fraction onto Se0_ens_-L1/Se0_ens_. Right: bar graph shows the normalized pathway weight for all direct, indirect, and combined direct/indirect sensory pathways onto the Se0_ens_-L1/Se0_ens_. Colors indicate different sensory modalities. Se0_ens_ mainly integrate mechanosensory information from ENS/pharynx and only to a small extent chemosensory inputs from ENS. (E) CaMPARI experiments: local stimulation of Piezo-expressing EG_post_ directly after feeding phase (yeast), but not after non-feeding phase (water), resulted in significantly increased neural activity of Se0 neurons. Data shown as boxplots. Performed statistical test: Mann-Whitney rank-sum test. (F) CaMPARI experiments: Se0 neurons in *Piezo*^*(*−*/*−*)*^ background showed reduced neural activity while ingesting yeast. Data shown as boxplots. Performed statistical test: Mann-Whitney rank-sum test. Abbreviations: Aorta_sens_, sensory neurons of the aorta; EG_ant/med/post_, esophageal ganglion (anterior, medial, and posterior); ENS_chemo/mechano_, enteric neuron (chemosensory, mechanosensory); ERM_motor_, esophageal ring musculature motor neuron; Ext_chemo/mechano_, external neuron (chemosensory, mechanosensory); GUS/Gustatory, gustatory neuron; HCG_sens/VGlut_, hypocerebral ganglion (sensory neuron, VGlut-positive neuron); IN_1_, interneuron 1; mNSC, median neurosecretory cell; Ph_mechano_, pharyngeal neuron (mechanosensory); Olfaction, olfactory neuron; Ph_motor_, pharyngeal motor neuron; PN, projection neuron; PVG_sens_, proventricular ganglia (sensory neurons); S_x_y, sensory neuron; Se0_ens_, enteric Se0 neuron; Somato, somatosensory neuron; TD_CO2/somato_, TD neuron (CO_2_-sensitive, somatosensory); Thermo, thermosensory neuron; VN, vagus nerve. See also Figures S6–S8 and Table S3.

It was unexpected to see that the different esophageal sensory neurons have such distinct monosynaptic output targets, high-lighted by the high degree of input from the EG_post_ to the Se0_ens_. To determine how the sensory-motor-modulatory system is organized in the entire feeding system, we determined all monosynaptic contacts between all sensory neurons and output targets of the ENS (Figures 6A and 6B). This revealed the putative physiological roles of the individual sensory neurons. For example, the HCG_sens_ and Aorta_sens_ strongly target the medial neurosecretory cells (mNSCs) and are likely involved with post-ingestive processes, such as sensing nutritive value, rather than with swallowing per se. It also revealed a second set of sensory neurons, in addition to the EG_post_, that makes strong contact with Se0_ens_ neurons, namely those from the PVG that innervate the most posterior part of the esophagus. From the perspective of Se0_ens_, nearly 40% of their total synaptic inputs originate from EG_post_ and PVG_sens_; from the perspective of EG_post_ and PVG_sens_, about 10%–12% of their synaptic outputs target Se0_ens_ (Figure 6B). Although the precise function of PVG_sens_ innervation is not known, it is remarkable that these sensory neurons are also located in a region that marks the final, irreversible stage of swallowing, as the food passes from the proventriculus into the midgut.

As pointed out earlier, the whole-larva STEM volume allows for the mapping of every monosynaptic connection from sensory neurons to the target cells at single-cell resolution, which we illustrate for Se0_ens_ (Figure S6). This single-cell connectivity map between the periphery and the brain provides insights into how variability and individuality is built into the connectivity pattern, since each of the six Se0_ens_, although viewed as a single cluster, has slightly different inputs and output patterns when analyzed at single-cell and synapse level.

All enteric sensory neurons, except for the Aorta_sens_ neurons, bifurcate in the periphery and enter the CNS bilaterally (Figure 6B). Comparing the connectivity pattern separately within the two halves did not show any striking qualitative differences between the left and right hemispheres (Figure S7). The bilateral analysis further strengthens the connectivity pattern, as it provides in essence a “*n* = 2” in the whole-larva STEM dataset in terms of connections within the CNS.

### Interneuronal paths enable multimodal sensory inputs onto Se0_ens_

The neuronal pathways presented so far represent monosynaptic connections. To determine how polysynaptic pathways connect the sensory and output neurons of the ENS, we turned to our previous analysis of the feeding circuit based on the whole CNS dataset (Figure 6C).^46,47,59,60^ We completely reconstructed all synaptic inputs to the Se0_ens_ in the whole CNS dataset and combined these with current analysis to identify the indirect sensory pathways to Se0_ens_ via interneurons.

Each interneuron has a distinguishing “sensory fingerprint” profile in terms of sensory composition, with differing degrees of shared and unique information, in targeting a single Se0_ens_ neuron (shown for Se0_ens_-L1; Figures 6C and S8A–S8C). The analysis was extended to all Se0_ens_ neurons by scaling the contribution of each direct and indirect sensory pathway. We first calculated the fraction of each sensory modality input to given interneurons and multiplied it with the fraction of input to each Se0_ens_ neuron. The sum of these indirect sensory pathway weights and the fraction of directly integrated sensory information predicts that the mechanosensory ENS_mechano_ and Ph/Ext_mechano_ are the predominant sensory inputs to the Se0_ens_, while ENS_chemo_ and gustatory inputs are integrated to a smaller extent (Figures 6D, S8D, and S8E). Thus, the vast majority of direct inputs to the Se0_ens_ are from mechanosensory inputs of the ENS; the major difference that occurs through integration of interneurons is a large increase in mechanosensory inputs from the pharyngeal and external sensory organs, including the gustatory neurons.

For functional validation, we focused on the *Piezo*-expressing EG_post_ neurons that have strong monosynaptic inputs to the Se0_ens_. We activated the EG_post_ with TrpA1 and monitored the activity of the Se0_ens_ with CaMPARI. This was done under two different nutrient conditions, one with water (low feeding) and one with yeast (high feeding). When presented with water, activation of EG_post_ did not result in a significant increase in Se0 activity. Critically, performing these experiments under yeast conditions resulted in significantly increased Se0 activity (Figure 6E). Se0_ens_ activity also increased with the application of a nutrient with higher viscosity (Figure S8D), and in Piezo^(−*/*−)^ animals, the induction of Se0_ens_ activity upon yeast feeding is no longer observed (Figure 6F). These data indicate that the neuronal response of Se0_ens_ is not solely due to mechanosensory input but also to a combined mechano-gustatory input and that mechanosensory input is necessary but not sufficient for activation of Se0_ens_ (Figures 6E and S8E). Taken together, the circuit architecture and functional analysis favor a mechanism by which the Se0_ens_ respond primarily to mechanosensory inputs from the EG_post_ that food has successfully moved through the esophagus but that their activity is also dependent on food quality such as taste and texture.

### Synapse placement within the core swallowing circuit

**Figure 7:**
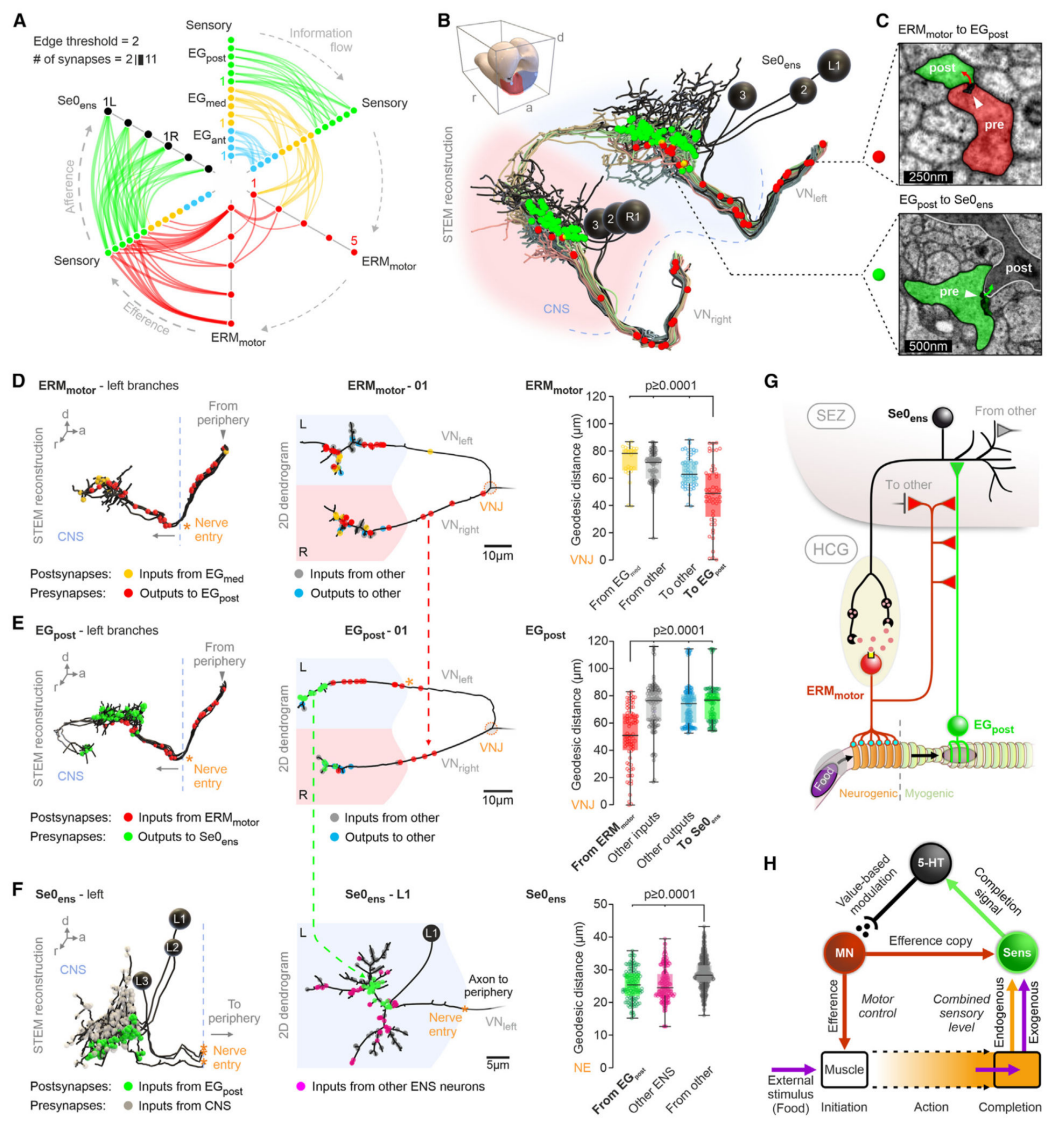
Action completion circuit of the core swallowing system (A) Radial information flow diagram of sensorimotor circuit for swallowing. (B) Three-dimensional STEM reconstruction of the elemental swallowing circuit in the CNS, showing all neurons from (A) and highlighting the synaptic connection from EG_post_ to Se0_ens_ (green) and from ERM_motor_ to EG_post_ (red). Se0_ens_ neurons are shown in black. (C) STEM slice showing a synaptic connection from ERM_motor_ to EG_post_ (top) and from EG_post_ to Se0_ens_ (bottom). (D) Three-dimensional STEM reconstruction (left) and two-dimensional dendrograms (middle) of ERM_motor_; axo-axonic connection from EG_med_ to ERM_motor_ (yellow) and ERM_motor_ to EG_post_ (red) are indicated. Synapse spatial position analysis (right) shows that ERM_motor_ to EG_post_ are located in front of all other output or input sites. Performed statistical test: one-way ANOVA. (E) Three-dimensional STEM reconstruction (left) and two-dimensional dendrograms (middle) of EG_post_; axo-axonic connection from ERM_motor_ to EG_post_ (red) and the axo-dendritic connection from EG_post_ to Se0_ens_ (green) are indicated. Synapse spatial position analysis (right) shows that EG_post_ input sites from ERM_motor_ are located prior to outputs onto Se0_ens_ neurons. Performed statistical test: one-way ANOVA. (F) Three-dimensional STEM reconstruction (left) and two-dimensional dendrograms (middle) of Se0_ens_-L1; the axo-dendritic connection from EG_post_ to Se0_ens_ (green) and from other enteric neurons to Se0_ens_ (magenta) are shown. Synapse spatial position analysis (right) shows that inputs from EG_post_ neurons show significantly lower geodesic distance, compared with other non-enteric inputs. Performed statistical test: one-way ANOVA. (G) Circuit architecture for sensory integration of EG_post_ neurons along spatially distinct regions of the esophagus during food swallowing. Food passage is detected by mechanoreceptive EG_post_ neurons, which then act on Se0 neurons to release serotonin. ERM_motor_ is modulated by serotonin, and ERM_motor_ is integrated onto the EG_post_ neurons. (H) Illustration of the action completion circuit for the food swallowing motor program. Abbreviations: EG_ant/med/post_, esophageal ganglion (anterior, medial, and posterior); ERM, esophageal ring musculature; ERM_motor_, ERM motor neuron; HCG, hypocerebral ganglion; MN, motor neuron; post, postsynaptic site; pre, presynaptic site; Sens, sensory neuron; Se0_ens_, enteric Se0 neuron; SEZ, subesophageal zone; VN, vagus nerve; vagus nerve junction. See also Figure S9.

The placement of synapses on the neurites is a key determinant for the neuronal response in a circuit.^61^ Therefore, we investigated the topographical organization of the synapses within the swallowing circuit at subcellular level. Aside from the intrasensory and intramotor connections, the core circuit flow map (Figures 7A–7C) has two key elements. One is an axo-axonic connection where the ERM_motor_ send synaptic outputs to the EG_post_ neurons (Figures 7D and 7E); these connections exist in the periphery as well as in the central neuropil, which is the site of synaptic integration. The second is an axo-dendritic connection from the EG_post_ to Se0_ens_ (Figures 7E and 7F). We calculated the geodesic distance for each synaptic input and output of each ERM_motor_ and EG_post_ neuron using the VN junction (VNJ) as collective origin (Figures 7D–7F, right most column and S9). This revealed that the axo-axonic connections from ERM_motor_ onto EG_post_ show the lowest geodesic distance, compared with all other inputs, and occur before the axodendritic contacts from EG_post_ to Se0_ens_. For Se0_ens_, the axo-dendritic inputs of EG_post_, together with the other enteric neurons, are closer to the anticipated locus of spike initiation (CNS entry) than synaptic sites of all other neurons presynaptic to Se0_ens_. Thus, we consider that the enteric inputs, in particular EG_post_, are likely to have a superordinate influence on the neural activity of Se0_ens_.

## Discussion

The seemingly simple act of swallowing is arguably the single most salient decision that an animal has to make. The motor system underlying swallowing is the ultimate “final common path”^62^ for feeding behavior, as it is the irreversible action where food is taken into the body. Our elucidation of a complete *Drosophila* swallowing circuit, through the first use of a whole-larva STEM volume, enabled the identification of all feeding relevant connections between the body and the brain at single-cell and synaptic resolution. Our findings raise and illuminate three key conceptual issues. One, how does an organism determine that a desired action has been completed? Two, how is the value or quality of a completed action encoded in terms of fulfilling a biological need? Three, how is the action reinforced when it is perceived to have a high biological value? In the following, we address each of these issues in the context of swallowing action (summarized in Figures 7G and 7H).

### A mechanosensory circuit for signaling when an action has been completed

There is a clear separation in the functional and anatomical organization of the esophagus along the foregut. The region underlying voluntary movement (neurogenic) triggers deglutition at the pharynx-esophagus junction, whereas the region for involuntary movement (myogenic) completes the peristaltic wave. Of the three clusters of esophageal sensory neurons, EG_ant_ and EG_med_ innervate just the neurogenic region and are involved in initiation and coordination of swallowing. By contrast, EG_post_ innervates just the myogenic region and does not have an active role in deglutition per se, but it rather senses whether food has passed through the esophagus. Since the EG_post_ expresses the mechanoreceptor Piezo and becomes active during feeding, we hypothesize that distension of the esophagus along the myogenic region provides the stimulus for EG_post_, which monitors if swallowing action has been successfully completed. In a different behavioral context involving a motor circuit for larval locomotion, a “mission accomplished” signal has been suggested that responds to contraction of a body segment muscle^63^; the source and target of this putative signal has not been identified.

### A serotonergic modulatory circuit for measuring the biological value of a completed action

Not all completed actions are equal in value or quality in terms of the degree to which they fulfill a biological need. Humans, for example, can make swallowing movements without food, but there is a big difference in the level of perceived satisfaction and metabolic consequence whether food is actually swallowed or not. Being able to distinguish these two would be valuable for an animal since future feeding action will depend on which of these events have occurred. The input signals onto the Se0_ens_ neurons may represent “quality control,” such as the nutrient value, of the substance that is being swallowed. The brain-wide analysis of polysynaptic sensory-to-Se0_ens_ integration revealed that all second- and third-order interneuron pathways to Se0_ens_ convey sensory information in multimodal combinations. Some of these interneurons form convergence pathways for ongoing (e.g., taste inputs) as well as stored sensory information, e.g., from mushroom body output neurons.^47,59^ The Se0_ens_ neurons may thus function as an integrating center or coincidence detector, in which both the food quality information (e.g., gustatory signaling through pharyngeal sensory organs) and an action completion signal (mechanosensory signaling through EG_post_) are combined to encode the biological value of the swallowing action.

### A motor circuit for swallowing: Enhancing actions that fulfill a biological need

Reinforcing or stabilizing a particular action could be achieved through increasing the strength, frequency, or duration of muscle contractions. During deglutition, serotonin acts by increasing the occurrence of peristaltic waves through the myogenic region of the esophagus. The 5-HT7-positive ERM_motor_ has synaptic outputs not only to the esophageal muscles but also to the esophageal sensory neurons (EG_post_) that connect to the Se0_ens_. The ERM_motor_ would thus initiate deglutition and at the same time send an efferent signal to the EG_post_, which monitors completion of deglutition. Furthermore, the synapse position of the axo-axonic ERM_motor_-to-EG_post_ relative to the axo-dendritic EG_post_-to-Se0_ens_ connections may function to prevent the influence of endogenous sensory signals from the esophagus, which is not caused by movement of food. Assuming an inhibitory nature of this efference copy connection, the summed sensory response onto Se0_ens_ would be perceived as an exogenous signal, i.e., passage of externally derived food through the esophagus. Efference copy has been used to describe how an animal distinguishes sensory information that arises from its own actions, compared with those in response to an environmental signal.^64–66^ For Se0_ens_, this information is combined with the nutrient value information of the swallowed substance via the multimodal sensory pathways onto Se0_ens_ in the CNS, which would guide future feeding actions through an elemental form of motor learning.

### Serotonin and ancient brain-body connections coordinating active movements

Stabilizing motor activity has been proposed to be a fundamental organizing principle underlying serotonin function.^67^ Work in cats^34^ showed that neural activity of certain serotonergic neurons that are activated by somatosensory and proprioceptive stimulation is associated with motor output activity, suggesting a facilitating effect of serotonin on motor function. In zebrafish, serotonergic neurons are involved in switching between flexible actions during swimming.^68,69^ In the leech, serotonin and mechanoreception in the gut are involved in switching between feeding and locomotive behaviors.^70–72^ Serotonergic neurons are also involved in controlling different aspects of feeding behavior in *Drosophila*.^10,73–75^ In *C. elegans*, enteric serotonergic neurons respond to food ingestion and modulate the feeding circuit.^76,77^ In *Aplysia*, the sensitization of the gill withdrawal reflex has three basic components (a mechanosensory neuron, a motor neuron, and a modulatory serotonergic neuron) whose coordinated activity is required for memory formation.^78,79^ What has been lacking in all these cases is the identity of the synaptic connections between the sensory, motor, and modulatory neurons within functional circuits. For example, despite decades of research, the identity and connectivity of the sensory neurons that provide monosynaptic or polysynaptic inputs to the serotonergic neurons utilized in the *Aplysia* gill withdrawal behavior remain elusive.^80,81^ An analogous but more complex circuit exists in songbird motor learning,^82,83^ in which the circuit utilizes dopamine rather than serotonin but where the essential features of the system can be discerned: the song motor system that connects to the auditory sensory system via efference copy, which is coupled to a dopaminergic reward system to reinforce a vocal motor pattern. In the fly swallowing circuit elucidated here, the motor circuit directly connects to a mechanosensory system, which is coupled to a serotonergic reward system that enhances the deglutition motor pattern.

Serotonin signaling has been implicated in motor function in the mammalian esophagus as well.^84,85^ The human esophagus also has distinct regions, with a proximal striated muscle region that is mainly controlled by motor neurons in the brainstem and a distal smooth muscle region that is controlled by central neurons in the medulla oblongata and peripheral neurons of the myenteric plexus.^86^ Despite the differences in the number of cell types as compared with the fly, it would be interesting to see if serotonin also monitors the completion of a biologically meaningful action such as swallowing or other vital activities in mammals.

### Resource Availability

#### Lead contact

Further information and requests for resources and reagents should be directed to and will be fulfilled by the lead contact, Michael J. Pankratz (pankratz@uni-bonn.de).

#### Materials availability

Newly generated fly lines are listed in the key resources table and are available from the lead contact upon request.

## Star⋆Methods

Detailed methods are provided in the online version of this paper and include the following:

- KEY RESOURCES TABLE
- EXPERIMENTAL MODEL AND SUBJECT DETAILS
  ○ Fly work
  ○ Fly lines and genotypes
  ○ Construction of plasmids and generation of *lexAop2-CaMPARI* transgenic fly line
- METHOD DETAILS
  ○ Dissection of semi-intact larva
  ○ Immunohistochemistry
  ○ Functional Imaging
  ○ Electrophysiological recordings
  ○ Optogenetic manipulation
  ○ Thermogenetic manipulation
  ○ Behavioral assays
  ○ EM reconstruction
- QUANTIFICATION AND STATISTICAL ANALYSIS

## Star ⋆Methods

### Key Resources Table

**Table:** 

REAGENT or RESOURCE	SOURCE	IDENTIFIER
Antibodies		
Chicken polyclonal anti-GFP	Abcam	Cat# ab13970; RRID:AB_300798
Rabbit polyclonal anti-5-HT	Sigma-Aldrich	Cat# S5545; RRID:AB_477522
Guinea pig polyclonal anti-Trhn	This study	N/A
Rabbit polyclonal anti-VGlut	Gift from H. Aberle^87^	N/A
Mouse monoclonal anti-elav	DSHB	Cat# Elav-9F8A9; RRID:AB_528217
Mouse monoclonal anti-22c10	DSHB	Cat# 22c10; RRID:AB_528403
Rabbit polyclonal anti-sNPF	Gift from J. Veenstra^88^	N/A
Mouse monoclonal anti-pros	DSHB	Cat# Prospero (MR1A), RRID:AB_528440
Goat polyclonal anti-mouse Alexa Fluor 405	Thermo Fisher Scientific	Cat# A-31553; RRID:AB_221604
Goat polyclonal anti-chicken Alexa Fluor 488	Thermo Fisher Scientific	Cat# A-11039; RRID:AB_2534096
Goat polyclonal anti-rabbit Alexa Fluor 633	Thermo Fisher Scientific	Cat# A-21071; RRID:AB_2535732
Goat polyclonal anti-guinea pig Alexa Fluor 633	Thermo Fisher Scientific	Cat# A-21105; RRID:AB_2535757
Goat polyclonal anti-mouse Alexa Fluor 633	Thermo Fisher Scientific	Cat# A-21052; RRID:AB_2535719
Phalloidin-TRITC	Sigma-Aldrich	P1951
Chemicals, peptides, and recombinant proteins		
All-trans retinal	Sigma-Aldrich	R2500
Serotonin-hydrochloride	Sigma-Aldrich	H9523
Copper(II) sulfate solution	Sigma-Aldrich	C2284
D(-)-fructose	Carl Roth	4981.1
Caffeine	Carl Roth	815.1
Sodium chloride	Thermo Fisher Scientific	10616082
Denatonium benzoate	Tokyo Chemical Industries (TCI)	D2124
Hydroxypropyl cellulose	Thermo Fisher Scientific	10723191
Deposited data		
STEM data	Peale et al.^52^/This study	https://doi.org/10.6084/m9.figshare.26510896
Experimental models: Organisms/strains		
*5-HT1A* ^*2A-Gal4*^	Kondo et al.^56^	N/A
*5-HT1B* ^*2A-Gal4*^	Kondo et al.^56^	N/A
*5-HT2A* ^*2A-Gal4*^	Kondo et al.^56^	N/A
*5-HT2B* ^*2A-Gal4*^	Kondo et al.^56^	N/A
*5-HT7* ^*2A-Gal4*^	Kondo et al.^56^	N/A
*52D06-Gal4*	Bloomington Drosophila	RRID:BDSC_38828
	Stock Center (BDSC)	
*30F10-Gal4*	BDSC	RRID:BDSC_49643
*Gr43a* ^*Gal4*^	BDSC	RRID:BDSC_ 93447
*Mef2-Gal4*	BDSC	RRID:BDSC_ 27390
*OK371-Gal4*	BDSC	RRID:BDSC_ 26160
*peb-Gal4*	BDSC	RRID:BDSC_80570
*Piezo-Gal4* ^*IIA*^	BDSC	RRID:BDSC_58771
*Piezo-Gal4* ^*III*^	BDSC	RRID:BDSC_59266
*Piezo* ^*Gal4.KI*^	BDSC	RRID:BDSC_78335
*Se0*_*ens*_*-Gal4* (29H01-Gal4)	BDSC	RRID:BDSC_47343
*Se0*_*ph*_ *-Gal4 (mn9-Gal4)*	McKellar et al.^26^	N/A
*Trhn-Gal4*	BDSC	RRID:BDSC_38389
*Trhn-lexA*	Alekseyenko et al.^89^	N/A
*VGlut-GAL4*	BDSC	RRID:BDSC_24635
*lexAop-CaMPARI*	This study	N/A
*UAS-5-HT7*	Kerr et al.^90^	N/A
*UAS-5-HT7-RNAi*	BDSC	RRID:BDSC_27273
*UAS-bPAC*	BDSC	RRID:BDSC_78788
*UAS-Brp∷GFP,UAS-CD8∷mCherry*	Urwyler et al.^91^	N/A
*UAS-Cam2.1*	BDSC	RRID:BDSC_6901
*UAS-CaMPARI*	BDSC	RRID:BDSC_58761
*UAS-CaMPARI2*	BDSC	RRID:BDSC_78316
*UAS-cAMPr*	Hackley et al.^92^	N/A
*UAS-Chrimson*	BDSC	RRID:BDSC_55135
*UAS-Epac1-camps*	BDSC	RRID:BDSC_25407
*UAS-GCaMP6f*	BDSC	RRID:BDSC_42747
*UAS-GCaMP6s*	BDSC	RRID:BDSC_42749
*UAS-GFP*	BDSC	RRID:BDSC_32184
*UAS-GtACR1*	BDSC	RRID:BDSC_92983
*UAS-myrGFP*	BDSC	RRID:BDSC_32197
*UAS-nSyb-GFP*	BDSC	RRID:BDSC_6921
*UAS-Piezo*	BDSC	RRID:BDSC_78336
*UAS-RFP*	BDSC	RRID:BDSC_27398
*UAS-Trhn-RNAi (Trhn-RNAi-1)*	Albin et al.^74^	N/A
*UAS-TrpA1*	BDSC	RRID:BDSC_26263
*VGlut-GAL80*	BDSC	RRID:BDSC_58448
*OrgR*	BDSC	RRID:BDSC_5
*Piezo* ^*KO*^	BDSC	RRID:BDSC_58770
Oligonucleotides		
Forward primer to amplify *CaMPARI* fragment: GTCGACCATGCTGCAGAACGAGCTT	This study	N/A
Reverse primer to amplify *CaMPARI* fragment: CTGATCAGCGAGCTCTAGCAT	This study	N/A
Recombinant DNA		
*pcDNA3-CaMPARI*	Fosque et al.^93^	Addgene plasmid #60421
*pJFRC19-13XLexAop2-IVS-myr∷GFP*	Pfeiffer et al.^94^	Addgene plasmid #26224
*pJFRC19-13XLexAop2-CaMPARI*	This study	N/A
Software and algorithms		
ImageJ	Schindelin et al.^95^	RRID:SCR_003070
SigmaPlot (12.0)	Grafiti LLC	RRID:SCR_003210
PRISM (9.0)	GraphPad	RRID:SCR_002798
Spike2 (7.20)	Cambridge Electronic Device	RRID:SCR_000903
ZEN 2.3 SP1 FP3 (black)	Zeiss	RRID:SCR_013672
CATMAID	Saalfeld et al.^96^	RRID:SCR_006278
Blender	Blender	RRID:SCR_008606
CaMPARI-analysis-Tool	This study	https://github.com/Pankratz-Lab/ImageJ-Scripts
Epac-analysis-Tool	This study	https://github.com/Pankratz-Lab/ImageJ-Scripts
cAMPr-analysis-Tool	This study	https://github.com/Pankratz-Lab/ImageJ-Scripts
Food intake-analysis tool	Schoofs et al.^10^	https://github.com/Pankratz-Lab/ImageJ-Scripts
GCaMP-recordings(cycle frequency)	This study	https://github.com/Pankratz-Lab/Spike2-Scripts

### Experimental Model And Subject Details

#### Fly work

All larvae were kept on 25 °C under 12 h light/dark cycle if not otherwise stated. For behavioral experiments 4 h egg collections were made on apple juice agar plates containing a load of yeast/water paste. After 48 h, larvae were transferred into vials (60 larvae per vial) containing standard cornmeal medium. For other experiments, e.g. functional imaging, electrophysiological recording and antibody staining, 4 h egg collection were made in vials with standard cornmeal medium with a spot of yeast/water paste and afterwards kept for four days on 25 °C. Only larvae for optogenetic stimulation were raised on fly food containing 150 μM all-trans retinal (Sigma-Aldrich, R2500) and kept under dark conditions.

#### Fly lines and genotypes

All larvae used for the experiments were 96±2 h old. The following *Drosophila melanogaster* lines were used (see also key resources table):

##### Driver lines

*5-HT1A*^*2A-Gal4*^, *5-HT1B*^*2A-Gal4*^, *5-HT2A*^*2A-Gal4*^, *5-HT2B*^*2A-Gal4*^, *5-HT7*^*2A-Gal4*56^ *52D06-Gal4* (BDSC #38828), *30F10-Gal4* (BDSC #49643), *Gr43a*^*Gal4*^ (BDSC #93447), *Mef2-Gal4* (BDSC #27390), *OK371-Gal4* (BDSC #26160), *peb-Gal4* (BDSC #80570), *Piezo-Gal4*^*IIA*^ (BDSC #58771), *Piezo-Gal4*^*III*^ (BDSC #59266), *Piezo*^*Gal4.KI*^ (BDSC #78335), *Se0*_*ens*_*-Gal4* (named “R29H01-Gal4 in,^97^ BDSC #47343), *Se0*_*ph*_*-Gal4* (named “*mn9*” in McKellar et al.^26^), *Trhn-Gal4* (BDSC #38389), *Trhn-lexA*,^89^ *VGlut-GAL4* (BDSC #24635).

##### Effector/reporter lines

*lexAop-CaMPARI* (for generation see below), *UAS-5-HT7*,^90^ *UAS-5-HT7-RNAi* (BDSC #27273), *UAS-bPAC* (BDSC #78788), *UAS-Brp::GFP*,*UAS-CD8::mCherry*,^91^ *UAS-Cam2.1* (BDSC #6901), *UAS-CaMPARI* (BDSC #58761), *UAS-CaMPARI2* (BDSC #78316), *UAS-cAMPr*,^92^ *UAS-Chrimson* (BDSC #55135), *UAS-Epac1-camps* (BDSC #25407), *UAS-GCaMP6f* (BDSC #42747), *UAS-GCaMP6s* (BDSC #42749), *UAS-GFP* (BDSC #32184), *UAS-GtACR1* (BDSC #92983), *UAS-myrGFP* (BDSC #32197), *UAS-nSyb-GFP* (BDSC #6921), *UAS-Piezo* (BDSC #78336), *UAS-RFP* (BDSC #27398), *UAS-Trhn-RNAi* (*Trhn-RNAi-1*,^74^), *UAS-TrpA1* (BDSC #26263), *VGlut-GAL80* (BDSC #58448).

##### Other lines

- *OrgR* (BDSC #5), *Piezo*^*KO*^ (BDSC #58770).
- The genotypes used in each figure are listed in Table S3.

#### Construction of plasmids and generation of *lexAop2-CaMPARI* transgenic fly line

Standard molecular biology methods were used and constructs were sequence verified prior to microinjection into fly embryos. Restriction enzymes and T4 DNA ligase were from New England Biolabs. PCR amplifications were performed with Q5 polymerase (New England Biolabs).

First *CaMPARI* coding sequence was PCR amplified from plasmid *pcDNA3-CaMPARI* (gift from Loren Looger & Eric Schreiter, Addgene plasmid #60421)^93^ with primers 5’-GTCGACCATGCTGCAGAACGAGCTT-3’ and 5’-CTGATCAGCGAGCTCTAGCAT-3’. The PCR product was subcloned into *pCRII-TOPO* vector (Invitrogen) resulting in plasmid *TOPO-CaMPARI*. Then, *myrGFP* from *pJFRC19-13XLexAop2-IVS-myr::GFP* (gift from Gerald Rubin, Addgene plasmid #26224)^94^ was removed by *Xho*I/*Xba*I digest and replaced with *Sal*I/*Xba*I fragment from *TOPO-CaMPARI* harboring *CaMPARI* coding sequence, generating plasmid *pJFRC19-13XLexAop2-CaMPARI*. Plasmid microinjections to generate two *lexAop-CaMPARI* fly lines (*P{y*^*+t7.7*^ *w*^*+mC*^*=13XLexAop2-CaMPARI}attP40* and *PBac{y*^*+*^ *w*^*+mC*^*=13XLexAop2-CaMPARI}VK00027*) were performed by BestGene Incorporated.

### Method Details

#### Dissection of semi-intact larva

Feeding 3^rd^ instar larvae were dissected in petri dishes coated with a two-component silicone elastomer (Wacker Chemical Corporation, Elastosil RT 601). Larvae were pinned down dorsal side up at the posterior and anterior end using sharp-etched tungsten needles (diameter: 40-60 μm). Larva was cut open longitudinally along the dorsal midline and thereafter the cuticle was cut transversely below the CPS with a micro scissors (Fine Science Tools, 15000-08). Interior organs like fat body, trachea or salivary glands were removed except for the CNS and CPS including the associated pharyngeal nerves and digestive tract up to the anterior midgut. This standard preparation of the *Drosophila* larva was used in all experiments which involve dissection of larvae, in the following termed semi-intact preparation. Any further dissections are documented separately in the individual experimental model and subject details sections.

#### Immunohistochemistry

Dissected larval brains were fixed for 1 h in paraformaldehyde (4 %) in 1⨯ phosphate-buffered saline (PBS), rinsed three times (20 min) with 1 % PBS-T (1 % Triton X-100 in 1⨯ PBS), and blocked in 1 % PBS-T containing 5 % normal goat serum (ThermoFisher) for 2 h. Primary antibody was added to the solution (for concentrations, see below). Brains rotated two nights at 4 °C. On the third day, after removing the primary antibody, larval brains were washed three times (20 min) with 1 % PBS-T, and additionally blocked in 1 % PBS-T containing 5 % normal goat serum for 30 min. Afterwards the secondary antibody was applied. Brains rotated two nights at 4 °C. After three times washing (20 min) with 1 % PBS-T, brains were dehydrated and cleared through an ethanol-xylene series and mounted in DPX Mountant (Sigma-Aldrich). Imaging was carried out using a Zeiss LSM 780 confocal microscope with LCI Plan-Neofluar 25⨯ / 0.8 Imm Korr DIC M27 or Plan-Apochromat 63⨯ / 1.4 Imm DIC objective (oil). For antibody staining of the *driver > GFP/myrGFP/nSyb-GFP*, the primary antibody was anti-GFP (1:500, chicken, Abcam, ab13970). Secondary antibody was anti-chicken Alexa Fluor 488 (1:500, goat, Invitrogen, A-11039). For 5-HT/Trhn staining, primary antibodies were anti-5-HT (1:1000, rabbit, Sigma-Aldrich, S5545) and anti-Trhn (1:250, guinea pig, generated by Thermo, immunogen sequence: DSFEEAKEQMRAFAESIQR), secondary antibodies were anti-rabbit Alexa Fluor 633 (1:500, goat, Invitrogen, A-21071) and anti-guinea pig Alexa Fluor 633 (1:500, goat, Invitrogen, A-21105). For VGlut staining, primary antibody was anti-VGlut (1:1000, rabbit, gift from Hermann Aberle). The secondary antibody was anti-rabbit Alexa Fluor 633 (1:500, goat, Invitrogen, A-21071). For *5-HT7 > GFP* and *5-HT7 > GFP, VGlut-Gal80* staining, primary antibodies were anti-GFP (1:500, chicken, Abcam, ab13970) and anti-elav (1:500, mouse, DSHB, Elav-9F8A9). Secondary antibodies were anti-chicken Alexa Fluor 488 (1:500, goat, Invitrogen, A-11039) and anti-mouse Alexa Fluor 633 (1:500, goat, Invitrogen, A-21052). For background/neuropil staining, primary antibody was anti-22c10 (1:500, mouse, DSHB, 22c10). 22c10 was deposited to the DSHB by Seymour Benzer and Nansi Colley. Secondary antibody was anti-mouse Alexa Fluor 405 (1:500, goat, Invitrogen, A-31553) or anti-mouse Alexa Fluor 633 (1:500, goat, Invitrogen, A-21052). For serotonin receptor expression analysis following primary antibodies were used: anti-GFP (1:500, chicken, Abcam, ab13970), anti-sNPF (1:1000, rabbit, gift from Jan Veenstra) or anti-pros (1:500, mouse, DSHB, Prospero (MR1A)). Accordingly, secondary antibodies were anti-chicken Alexa Fluor 488 (1:500, goat, Invitrogen, A-11039) and anti-rabbit Alexa Fluor 633 (1:500, goat, Invitrogen, A-21071) or anti-mouse Alexa Fluor 633 (1:500, goat, Invitrogen, A-21052). For occasional F-actin staining, we used the conjugated fluorescent Phalloidin-TRITC (1:1000, Sigma-Aldrich, P1951).

For the 5-HTR expression analysis of the enteric nervous system, all five 5-HTR Gal4 lines^56^ were crossed with three different GFP reporter lines (GFP, myr-GFP, Cam2.1) for all three enteric ganglia (EG, HCG and PVG), the larval endocrine organ (RG) and the midgut (MG). In the immunohistochemical analysis the GFP reporter stains were combined with VGlut staining for HCG (ERM_motor_), sNPF stains for PVG (PVG_mod_) and pros stains for midgut (EEC) to identify the different cell/neuron types (Figure S3). For midgut antibody stainings larvae were fed on yeast containing 4% Copper(II) sulfate solution (Sigma-Aldrich, C2284) to mark the copper cells of the midgut as a landmark (Figure S3). Based on the double antibody staining and morphological features, the number of cells were counted for each single image according to their cell/neuron type in the expressed structure, except for the pharyngeal/midgut muscles and larval endocrine organ (RG). For the statistical analysis, the mean values (including the standard deviation and number of analyzed structures) were calculated and listed in Tables S1 and S2.

#### Functional Imaging

For Calcium-imaging by an integrator, we used CaMPARI^93^ and CaMPARI2.^98^ In the experiments with *peb > CaMPARI2* and *Piezo*-^*Gal4.KI*^ *> CaMPARI2* a starved larva (≥ 30 min starvation time) was placed on either a water agar plate (non-fed condition) or a water agar plate coated with yeast (fed condition). 405 nm UV light (Thorlabs, M405L2) connected to a LED controller (Thorlabs, LEDD1B) was positioned 12 cm above the larva and illuminated at max intensity for 2 min. Afterwards the larval esophagus was dissected and put onto a poly-L-lysine-coated coverslip and covered with 1⨯ PBS for imaging at low Ca^2+^ conditions. EG neurons with their dendrites covering the esophagus were imaged. For the *CaMPARI* experiments of Se0 neurons, we used *Trhn/Se0*_*ens*_*/Se0*_*ph*_ *> CaMPARI*. In gustatory experiments the larva was placed in a well of a Terasaki plate (Greiner, 659180) filled with 20 μl 10 % yeast (Uniferm)-/ 1 M fructose (Carl Roth, 4981.1)-/ 20 mM caffeine (Carl Roth, 815.1)-/ 10 mM denatonium (TCI, D2124)-/ 2 M NaCl-solution (Thermo Fisher Scientific, 10616082, dissolved in tap water)/ tap water. Or wells were filled with 20 μl 0.5 M fructose solution at different hydroxypropyl cellulose (HPC, Thermo Fisher Scientific, 10723191) concentration for mechanical stimulation. 405 nm UV light was positioned 12 cm above the larva and illuminated at max intensity for 30 s after a 2 min perception period. Afterwards the larval brain was dissected and placed onto a poly-L-lysine-coated coverslip and covered with 1⨯ PBS for imaging at low Ca^2+^ conditions. The SEZ region with the Se0 neurons was imaged. For the CaMPARI experiments of Se0 neurons while activating EG_post_ neurons, we used the genotype *Piezo*^*III*^*-Gal4 > UAS-TrpA1; Trhn-LexA > lexAop-CaMPARI*. Prior to the experiment, larvae were starved (≥ 30 min starvation time) and transferred on either a water agar plate or water agar plate coated with yeast for 2 min. Afterwards the larvae were dissected. A custom-made heating device for local thermal application was positioned close to the EG_post_ neurons without covering the CNS. A 405 nm UV light was positioned 6 cm above the semi-intact larvae. Simultaneously the larvae were illuminated at max intensity and thermal stimulus of 18 °C (no activation) or 32 °C (activation) was applied to EG_post_ neurons for 2 min. After this time period the brain was dissected and positioned on a poly-L-lysine-coated coverslip with 1⨯ PBS for imaging at low Ca^2+^ conditions. The Se0 neurons in the SEZ were imaged. All images were acquired using a ZEISS LSM 780 Laser scanning microscope with LCI Plan-Neofluar 25 ⨯ / 0.8 Imm Korr DIC M27. For quantification, intensity of the red fluorescence to the intensity of the green fluorescence ratios of single cells were analyzed with a custom-made script for FIJI (ImageJ; https://github.com/Pankratz-Lab), and the mean per animal was calculated (each cell was analyzed and mean calculated). Obtained data was then statistically analyzed and plotted with SigmaPlot (version 12) software using the Mann–Whitney rank-sum test.

For GCaMP-recordings, the genotypes *Se0*_*ens*_ *> GCaMP6f, Mef2 > GCaMP6f, Mef2 > GCaMP6s*,*RFP* and *30F10 > GCaMP6f* were used. To record the ERM and neural activity of ERM_motor_ in the HCG, dissected foregut and ENS of a larva based on semi-intact preparation was positioned on a poly-L-lysine-coated coverslip and covered with 18 μl saline.^99^ After 3 min initial recording 2 μl 10^-6^ M 5-HT solution was added to obtain a final experimental concentration of 10^-7^ M 5-HT. For recordings of the Se0_ens_ in the SEZ, we used semi-intact preparation with intact anterior region which were placed in a petri-dish coated with silicone elastomer. To reduce movement artifacts, the larval CNS was embedded in low-melting agarose. Feeding status was initiated by placing a small piece of yeast in front of the larval mouth cavity. Images were acquired with a Zeiss LSM 780 laser scanning microscope as time series with a scan speed of approximately 50 ms (~20 Hz) using a Zeiss LCI Plan-Neofluar 25⨯ / 0.8 Imm Korr DIC M27 objective dipped in the saline solution. For quantification, recordings were analyzed using the software Zen 2012 (Zeiss) and a custom-made script for Spike2 (Cambridge Electronic Design; https://github.com/Pankratz-Lab) to measure cycle frequency and completion rate for each experiment. The completion rate is the percentage of induced contraction waves in the myogenic region which successfully conveyed into the neurogenic region representing a complete esophageal peristalsis.

For cAMP-level measurement using the genotypes *OK371 > Epac1-camps*, larva was dissected and a semi-intact preparation was positioned on a poly-L-lysine-coated coverslip with 20 μl saline^99^ or fresh prepared 10^-7^ M 5-HT solution (solved in saline). 3 min after 5-HT application the ERM-MNs in the HCG were scanned. All images were acquired using a ZEISS LSM 780 Laser scanning microscope with LCI Plan-Neofluar 25 ⨯ / 0.8 Imm Korr DIC M27. For quantification, cyan fluorescence to yellow fluorescence red ratios for individual cells were measured with a custom-made script for FIJI (ImageJ; https://github.com/Pankratz-Lab), and the mean was calculated per animal (each cell was analyzed and a mean build). Animal means (including standard error of mean) were then analyzed and plotted with SigmaPlot (version 12) software.

For cAMP-level measurement using the genotypes *VGlut > cAMPr*, larva was dissected and a semi-intact preparation was positioned on a poly-L-lysine-coated coverslip covered with 20 μl saline^99^ or fresh prepared 10^-7^ M 5-HT solution (solved in saline). Imaging time series lasted 600 s in which each 30s an image was scanned. All images were acquired using a ZEISS LSM 780 Laser scanning microscope with LCI Plan-Neofluar 25⨯ / 0.8 Imm Korr DIC M27. For quantification, green fluorescence intensity for individual cells were measured with a custom-made script for FIJI (ImageJ; https://github.com/Pankratz-Lab). Mean of all analyzed cells (including standard error of mean) for each time point was analyzed and plotted with SigmaPlot (version 12) software.

#### Electrophysiological recordings

For extracellular recordings, semi-intact larva preparation was used to expose the pharyngeal nerve, e.g. vagal nerve (VN), the eye-antennal disk was removed. The nerve was insulated with a paraffin-petroleum jelly mixture. Neural activity was measured using custom made silver wire electrodes connected to an amplifier/signal conditioner system (Model MA 102&103, Neuroscience Electronics Laboratory, University of Cologne). All recorded signals were amplified (5,000 fold) and filtered (0.1–3 kHz). Recordings were sampled at 20 kHz. Data was acquired with Power 1401 mk II A/D board and Spike2 software (Cambridge Electronic Design). To measure only afferent signals, the CNS was removed after establishing the nerve recording.

#### Optogenetic manipulation

For optogenetic manipulation of neuronal activity, two effector lines *UAS-GtACR1*^100^ for inhibition and *UAS-Chrimson*^101^ for excitation were used in the respective experiments. As mentioned, larvae were bred on standard cornmeal medium containing 150μM all-trans retinal. Activation of these light-gated channels was induced by illumination at 530 nm (Chrimson) with a laser LED (Thorlabs, M530L3) or 625 nm (GtACR1) with a laser LED (Thorlabs, M625L3). In electrophysiological experiments the laser LEDs were attached to an optical fiber system (Thorlabs) to stimulate/inhibit specific regions of the ENS. For behavioral assays, the laser LED was mounted onto a collimated lens to illuminate the experimental area. LEDs were controlled via an A/D board (Power 1401 mk II, Cambridge Electronic Design) which was connected to voltage-controlled LED power supply (Thorlabs, LEDD1B). Stimulus timing and duration were set by protocols in Spike2 software (Cambridge Electronic Design). All optogenetic experiments were performed in darkness.

#### Thermogenetic manipulation

For thermogenetic manipulation of neuronal activity, *UAS-TrpA1*^102^ for excitation was used in respective experiments. Local thermal stimulus was applied with a custom-made heating device controlled by an A/D board.^10^ The local temperature of a tissue was shifted to 18 °C for non-activating or shifted to 32 °C for activating the respective TrpA1 expressing neuronal tissue.

#### Behavioral assays

For short-term food intake assay, only feeding third instar larvae (96±2 h) were used. Apple juice agar plates were prepared with a spot of colored yeast paste in the middle of the plate. For optogenetic experiments, the apple juice agar plate was coated with a thin layer of colored yeast to ensure activation of the effector. Before the experiment plates were placed at RT for 2 h. After 30 min starvation, five larvae were transferred on top of colored yeast paste for 5 or 20 min. Subsequently, larvae were transferred into a cell strainer and washed with 65 °C hot water. Killed larvae were then transferred onto glass slides for photo documentation and analyzed with FIJI software (ImageJ; https://github.com/Pankratz-Lab) by a custom written analysis macro, which determined the percentage of the colored surface of the intestinal system compared to body surface of the larva.

To monitor the motility of the larval foregut, videos of semi-intact larvae were recorded using a camera (Logitech, Quickcam Pro 9000 or Kurokesu, C1 Pro) mounted to a microscope (Zeiss, Stemi-2000C). Dark-field microscopy was used to improve visibility. VirtulDub or Spike2 was used as capturing software. For pharmacological experiments, a well was filled with 45 μl of saline solution and additional 5 μl of varying 5-HT solutions. In control experiments 5 μl of saline solution were instead applied. Optogenetic experiments were performed in a silicone elastomer-coated petri dish filled with saline solution. For optogenetic stimulation of EG_ant/med/post_ neurons, the PVG of the semi-intact larva was removed. Recorded videos were manually analyzed three times to determine the mean activity of ERM as peristaltic waves per minute. Only complete peristaltic waves from pharynx to proventriculus counted as activity.

#### EM reconstruction

Neuron reconstruction was done on a STEM (scanning transmission electron microscopy) volume of a whole first instar larva; the technical details of its generation are described separately in Peale et al.^52^ All reconstructions were made in a modified version of CATMAID (http://www.catmaid.org,^96^). For reconstructing a neuron, a specific neurite in a section of the STEM data set was identified and a neuronal three-dimensional skeleton including the synaptic active zones and synaptic partners was manually generated. We identified all enteric neurons, Se0 neurons and specific pharyngeal neurons by reconstruction of all axons passing through the frontal nerve junction (FNJ) originating either in the CNS or ENS. We reconstructed all neurons to completion (tracing 100% and at least 95% reviewed). For the Se0 neurons, as modulatory output neurons,^47^ all membrane fusion sides of CCVs (clear core vesicles) were marked as connectors without synaptic partners. To determine the putative downstream targets of Se0 neurons all tissues innervated by the ENS, e.g. foregut, midgut, ring gland and garland cells were reconstructed. For identified motor neurons, the innervated muscles were reconstructed and the neuromuscular junctions (NMJs) marked as connectors targeting the respective muscle. For identified neuropeptidergic neurons, the number of cytoplasmic DCVs (dense core vesicles) was determined.

### Quantification and Statistical Analysis

All ImageJ and Spike2 scripts used to analyze behavioral and physiological datasets are available at https://github.com/Pankratz-Lab.

All statistical analyses were carried out in SigmaPlot (12.0) or PRISM 9.0 (GraphPad). All performed statistical tests, number of replicates and statistical significance values of represented data are reported in the corresponding figure, figure legends and in the corresponding section of the method details. In all boxplots shown in figure and supplementary figure, the solid line depicts the median; the upper and lower boundary of the box depict the first and third quantiles of the data set, respectively. Whiskers indicate 5% and 95% confidence level. Individual data points of the box plots are included as circles. All measurements were taken from distinct samples.

To determine the influence that a particular sensory modality exerts on the Se0_ens_, we used the pathway weight which quantifies the relative overall influence of one neuron onto another through all linked synaptic pathways.^103^ Direct sensory pathway scores are the normalized synaptic fraction of each sensory neuron onto Se0_ens_. For computing indirect sensory pathways, we created one adjacency matrix of the synaptic fraction for each interneuron connected to Se0_ens_ (normalized to the total number of synaptic inputs onto each Se0_ens_). In a second adjacency matrix, we calculated the synaptic fraction of each sensory neuron onto these interneurons (normalized to total number of synaptic inputs onto the interneurons). To obtain all indirect sensory pathway scores, the normalized synaptic fraction for each “sensory neuron to interneuron” connection was multiplied by the normalized synaptic fraction of the respective “interneuron to Se0_ens_” connection. To calculate the full pathway weight for a sensory modality, we summed all direct and indirect pathways scores of the sensory neurons belonging to one category.

For the connectivity symmetry analysis of the whole larva STEM volume, for each neuron cluster of the ENS the percentage of incoming and outgoing synaptic budget for the left and right brain hemisphere was calculated. The asymmetry index (AI) is calculated from the formula AI = [(LL-RR)/(RR+LL)] x 100% for the ipsilateral connectivity and AI = [(LR-RL)/(RL+LR)] x 100% for the contralateral connectivity. Correlation of ipsi- and contralateral connectivity for incoming and outgoing synaptic budget was tested using the Spearman’s rank correlation test.

## Supplementary Material

Document S1. Figures S1–S9 and Tables S1–S3.

Document S2. Article plus supplemental information.

## Data Availability

STEM data sets of this study are publicly available as of the date of publication. DOI is listed in the key resource table. Raw microscopy, behavioral and experimental data will be supplied upon request by the lead contact, Michael J. Pankratz (pankratz@unibonn.de). Any original code that is part of the used analysis module has been deposited at GitHub and is publicly available as of the date of publication. DOIs are listed in the key resources table. Any additional information required to re-analyze the data reported in this paper is available from the lead contact upon request.

## Highlights

- STEM reconstruction of the entire enteric nervous system at the synaptic level
- Piezo-mechanoreceptive neurons convey completed swallowing action to the brain
- Central serotonergic neurons evaluate the biological valence of swallowed food
- Serotonin modulates swallowing through motor neurons driving esophageal peristalsis

## In brief

Using a whole-animal serial transmission electron microscopy dataset, Schoofs et al. provide a complete reconstruction of a sensorimotor circuit underlying the *Drosophila* swallowing system. Through the vagus nerve, serotonergic neurons rate food based on mechanosensory and chemosensory information and modulate esophageal peristalsis.
