# Supplementary material for: Serotonergic modulation of swallowing in a complete fly vagus nerve connectome
Source: Curr Biol. Author manuscript; Available in PMC 2024 Nov 19. (PMC7616834; doi:10.1016/j.cub.2024.08.025)
Supplement: Document S1. Figures S1–S9 and Tables S1–S3. [file EMS200071-supplement-Document_S1__Figures_S1_S9_and_Tables_S1_S3__.pdf]

Current Biology, Volume *34*

Supplemental Information

Serotonergic modulation of swallowing

in a complete fly vagus nerve connectome

Andreas Schoofs, Anton Miroschnikow, Philipp Schlegel, Ingo Zinke, Casey M. Schneider-Mizell, Albert Cardona, and Michael J. Pankratz

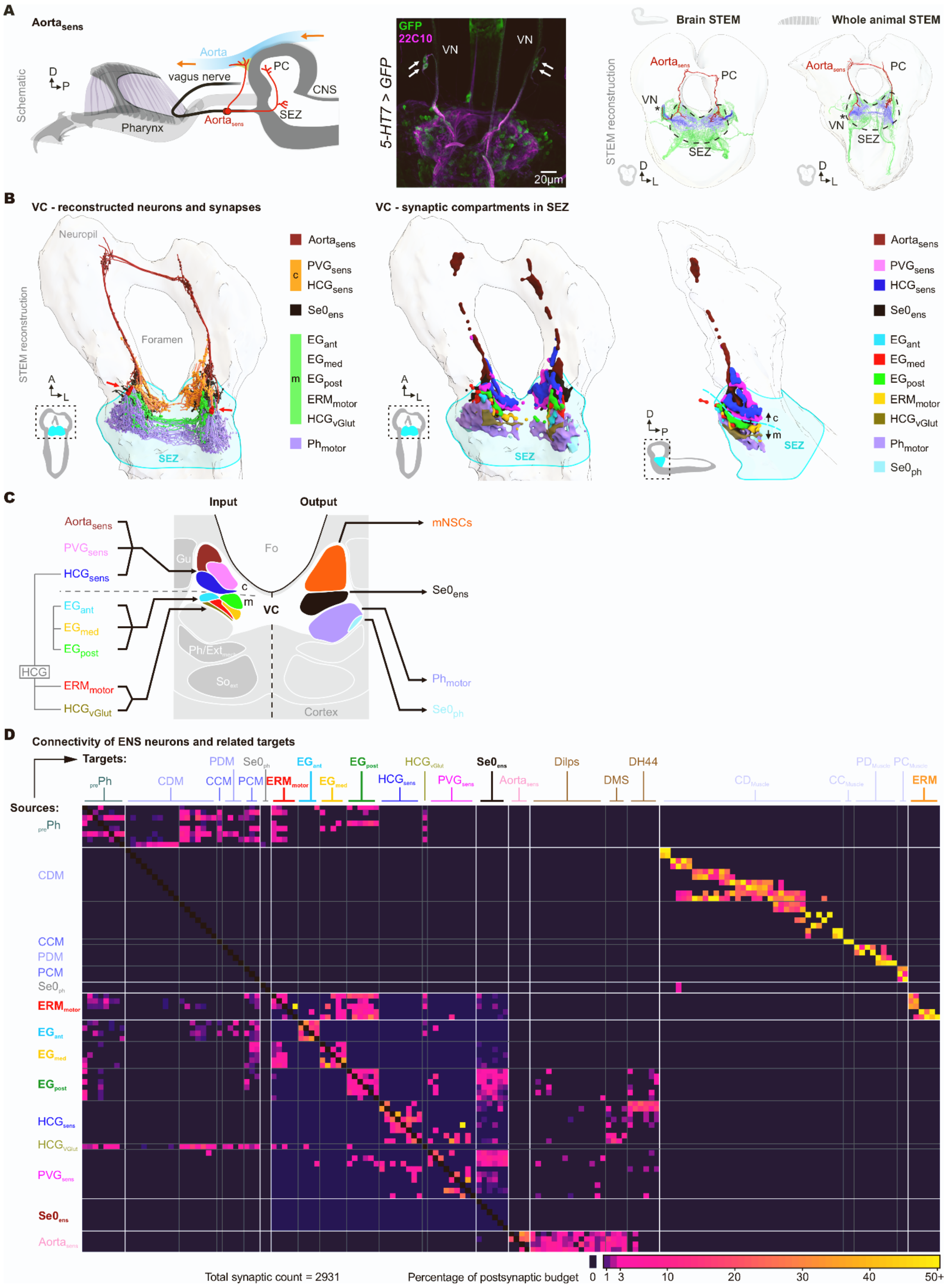

Figure S1: Reconstructed vagus center connectome of *Drosophila* larva. Related to Figure 1

(A) Left: schematic drawing of two Aorta$_{sens}$ neurons which have their receptive field on the aorta and have synaptic connections to the protocerebrum and the mNSCs (Hückesfeld et al., 2021; Schlegel et al., 2016). Middle: antibody staining of 5-HT7 > GFP showing the soma of the Aorta$_{sens}$ neurons in the VN. Right: three-dimensional illustration of the reconstructed Aorta$_{sens}$ neurons in the whole brain and whole animal STEM-volume.

(B) Left: three-dimensional illustration of the reconstructed neurons including the synapses for the larval VC. Red arrow

marks the VN entry site. Right: three-dimensional illustration of the relevant synaptic compartments. Note the distinct chemo- (c) and mechano-sensory (m) compartments.

(C) Illustration of the synaptic input (sensory) and synaptic output (motor/modulatory) compartments of the larval VC in the SEZ.

(D) Heat-map showing the connectivity of ENS neurons and related targets, including the pharyngeal motor system. Note that $Se0_{ph}$ does not receive synaptic inputs from enteric neurons, but from pharyngeal sensory neurons, which are not part of this work.

Abbr.: $Aorta_{sens}$ - sensory neurons of the aorta, CC_{muscle} – cibarial constrictor muscle, CCM - CC_{muscle} motor neurons, CD_{muscle} – cibarial dilator muscle, CDM - CD_{muscle} motor neurons, DH44 – diuretic hormone 44, Dilps – *Drosophila* insulin-like peptide, DMS - drosomyosuppresin, $EG_{ant/med/post}$ – esophageal ganglion (anterior, medial, posterior), ENS - enteric nervous system, ERM – esophageal ring musculature, ERM_{motor} – ERM motor neuron, $Ext/Ph_{mechano}$ - external/pharyngeal mechanosensory afference, Fo - foramen, Gu - gustatory afference, $HCG_{sens/VGlut}$ – hypocerebral ganglion (sensory neurons, VGlut-positive neuron), mNSCs - medial neurosecretory cells, PC - protocerebrum, PC_{muscle} – pharyngeal constrictor muscle, PCM - PC_{muscle} motor neurons, PD_{muscle} – pharyngeal dilator muscle, PDM - PD_{muscle} motor neuron, Ph_{motor} – pharyngeal motor neurons, ${}_{Pre}Ph$ – pharyngeal premotor neuron, PVG_{sens} – proventricular ganglion (sensory neurons), $Se0_{ens}$ – enteric Se0 neurons, $Se0_{ph}$ – pharyngeal Se0 neuron, So_{ext} – external somatosensory afference, SEZ - subesophageal zone, VC - vagus center, VN - vagus nerve.

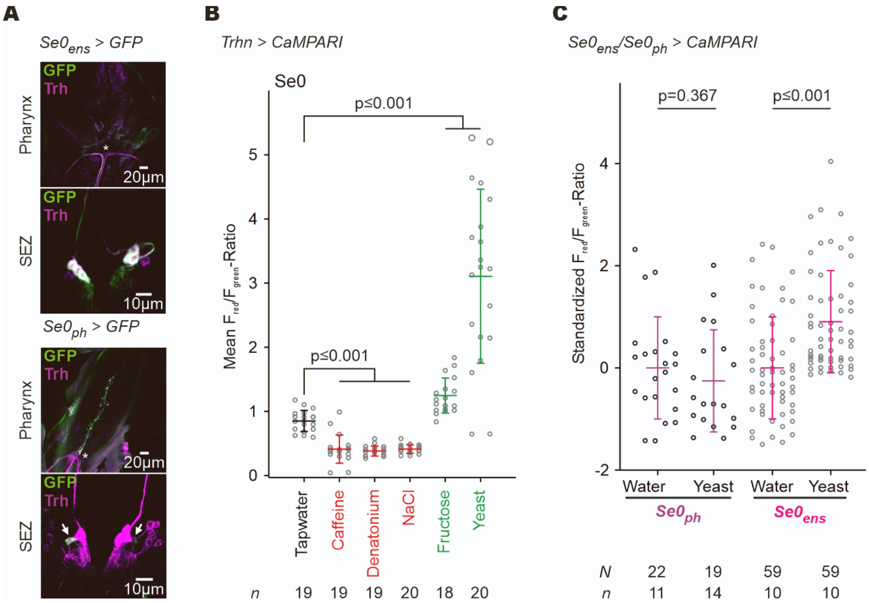

Figure S2. $Se0_{ph}$ and $Se0_{ens}$ are morphologically and functionally different subclusters. Related to Figure 2

(A) Antibody staining of *$Se0_{ens}$ > GFP* and *$Se0_{ph}$ > GFP*. The Gal4 driver lines show distinct expressions in the two Se0 subclusters. The three $Se0_{ens}$ neurons project into the entire ENS via VN_{ens} (indicated by asterisk). The one $Se0_{ph}$ neuron projects to pharynx via VN_{ph} (indicated by asterisk).

(B) CaMPARI-experiments showed that Se0 neurons respond with a decrease in neural activity after presentation/ingestion of aversive nutrients, e.g. caffeine, denatonium and NaCl (marked red). Otherwise attractive nutrients, like fructose and yeast, resulted in increased neural activity (marked green, data shows mean and ±SE). Performed significance test: Mann-Whitney rank sum test.

(C) CaMPARI-experiments of $Se0_{ph}$ and $Se0_{ens}$ neurons revealed that only $Se0_{ens}$ neurons showed increased neural activity upon ingestion of attractive nutrients but not $Se0_{ph}$ neurons. Data is shown as scatter plots (gray) including mean (colored line) and standard error (colored whiskers). Performed significance test: Mann-Whitney rank sum test.

Abbr.: $Se0_{ens}$ - enteric Se0 neurons, $Se0_{ph}$ - pharyngeal Se0 neurons, SEZ - subesophageal zone.

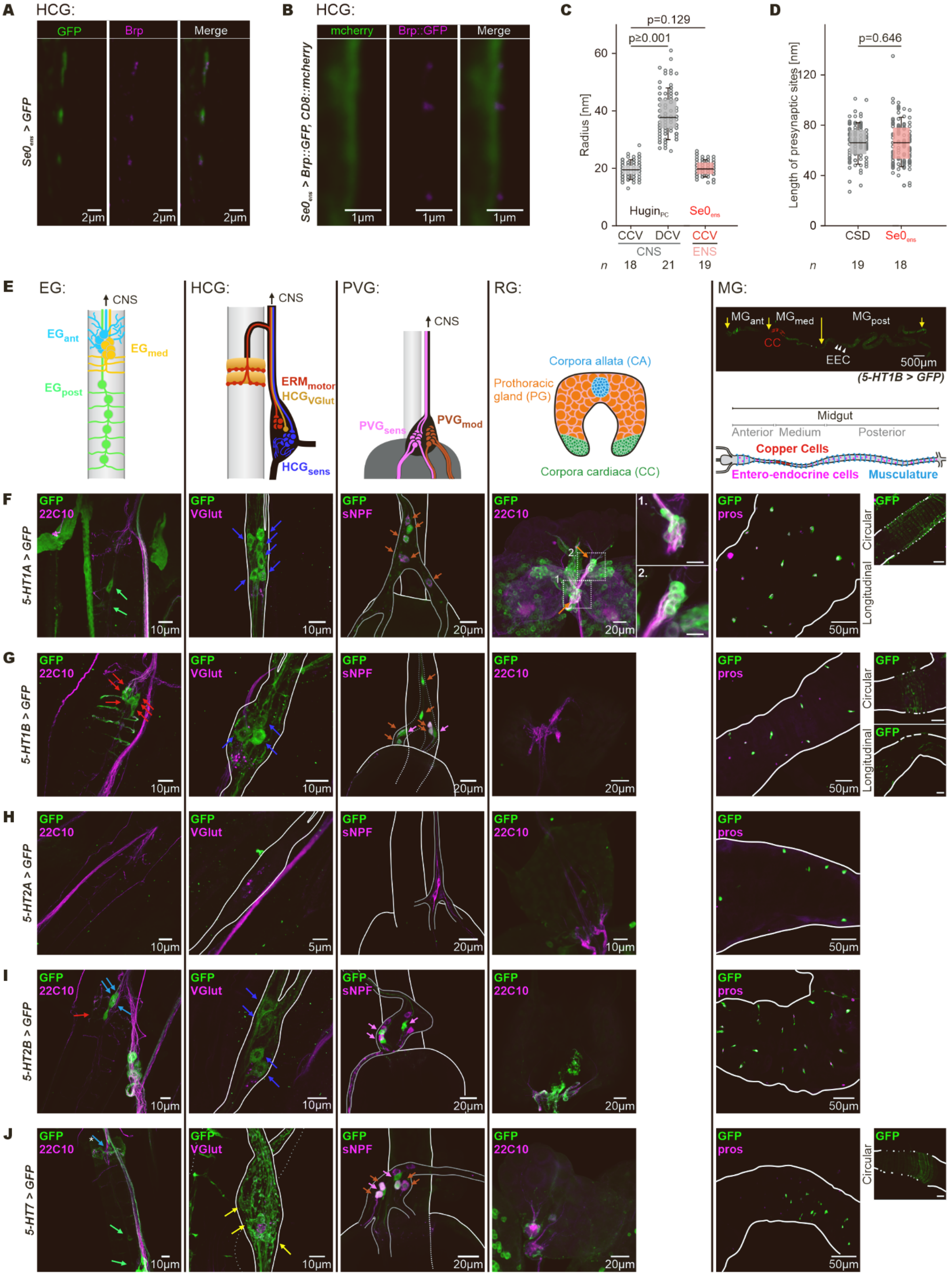

Figure S3. Peripheral active zones of Se0$_{ens}$ neurons and serotonin receptor expression analysis in the ENS. Related to Figure 2 and Table S1,S2

(A) Antibody staining of Se0$_{ens}$ > *GFP* against serotonin (5-HT) and *Bruchpilot* (*Brp*) indicating that peripheral active zones of serotonergic Se0$_{ens}$ neurons share structural similarities with presynaptic sites in the CNS.

(B) Antibody staining of Se0$_{ens}$ > *Brp::GFP, CD8::mcherry* indicating that peripheral active zones of serotonergic Se0$_{ens}$ neurons share structural similarities with presynaptic sites in the CNS.

(C) Box plots showing the radius of CCV (clear core vesicles) and DCV (dense core vesicles) for Hugin$_{PC}$ (grey, obtained from brain STEM volume) in the CNS compared to the radius of CCV of Se0$_{ens}$ (red, obtained from whole animal STEM volume) in the ENS. Note that there is no significant difference between the CCVs of Hugin$_{PC}$ and Se0$_{ens}$.

(D) Comparison between the length of peripheral active zones of Se0$_{ens}$ (obtained from whole animal STEM volume) and central presynaptic sites of the serotonergic CSD neuron (obtained from brain STEM volume). There is no significant difference in length between the central presynaptic sites and peripheral active zones.

(E) Schematic drawings of the ganglions of the ENS including their different neuron types (colored), larval endocrine organ (rind gland) and midgut which were analyzed for their expression of serotonin receptors.

(F-J) Fluorescence images show the GFP-expression for the serotonin receptors 5-HT1A (**F**), 5-HT1B (**G**), 5-HT2A (**H**) , 5-HT2B (**I**) and 5-HT7 (**J**) in ENS, RG and MG of *Drosophila melanogaster*. To identify the different cell types in the ENS or MG additional antibody stainings against VGlut (motor neurons), sNPF (modulatory neurons), pros (entero-endocrine cells) or 22C10 (neurites) were included. Colored arrows mark identified cell bodies of neurons.

Abbr.: CC - copper cells, CCV - clear core vesicles, DCV - dense core vesicles, EEC - entero-endocrine cells, EG$_{ant/med/post}$ – esophageal ganglion (anterior, medial, posterior), ENS - enteric nervous system, ERM$_{motor}$ – esophageal ring muscle motor neuron, HCG$_{sens/VGlut}$ – hypocerebral ganglion (sensory neuron, VGlut-positive neuron), MG$_{ant/med/post}$ - midgut (anterior, medial, posterior), PVG$_{mod/sens}$ –proventricular ganglion (modulatory neuron, sensory neuron), RG – ring gland.

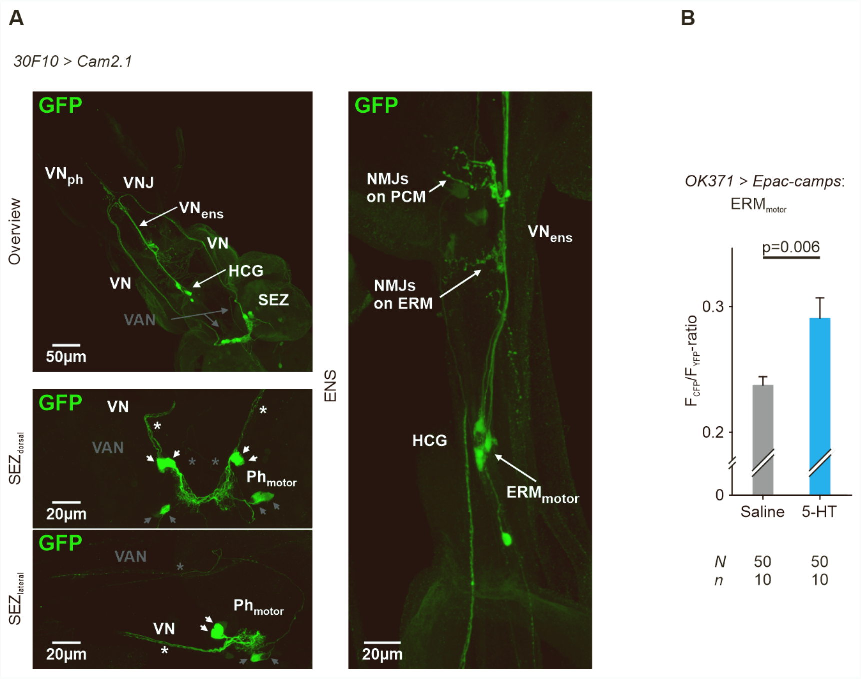

Figure S4. Expression pattern of *30F10-Gal4* and application of serotonin increases intracellular cAMP-level in ERM$_{motor}$. Related to Figure 3 and 4.

(A) *30F10 > Cam2.1* shows expression three different types of feeding-related motor neuron types: 1) ERM$_{motor}$ which are located in HCG, 2) Ph$_{motor}$ (white arrows) projecting through the VN (white asterisks) which innervate via VN$_{ph}$ the CDM and via VN$_{ens}$ the PCM and 3) Ph$_{motor}$ (gray arrows) projecting through the VAN (gray asteriks) which innervate the PDM.

(B) The cAMP-reporter (Epac1-camps) showed an increased cAMP-level in ERM$_{motor}$ after serotonin treatment (10^{-7}M) using the Gal4 driver line *OK371*. Data shows mean ±STD. Performed significance test: Mann-Whitney rank sum test.

Abbr.: cAMP – cyclic adenosine monophosphate, ERM - esophageal ring musculature, ERM$_{motor}$ - esophageal ring musculature motor neuron, HCG - hypocerebral ganglion, NMJ -neuromuscular junction, PCM - pharyngeal constrictor musculature, PDM - pharyngeal dilator musculature, Ph$_{motor}$ - pharyngeal motor neurons, SEZ - subesophageal zone, VAN - ventral arm nerve, VN - vagus nerve, VN$_{ens/ph}$ - enteric/pharyngeal vagus nerve, VNJ - vagus nerve junction.

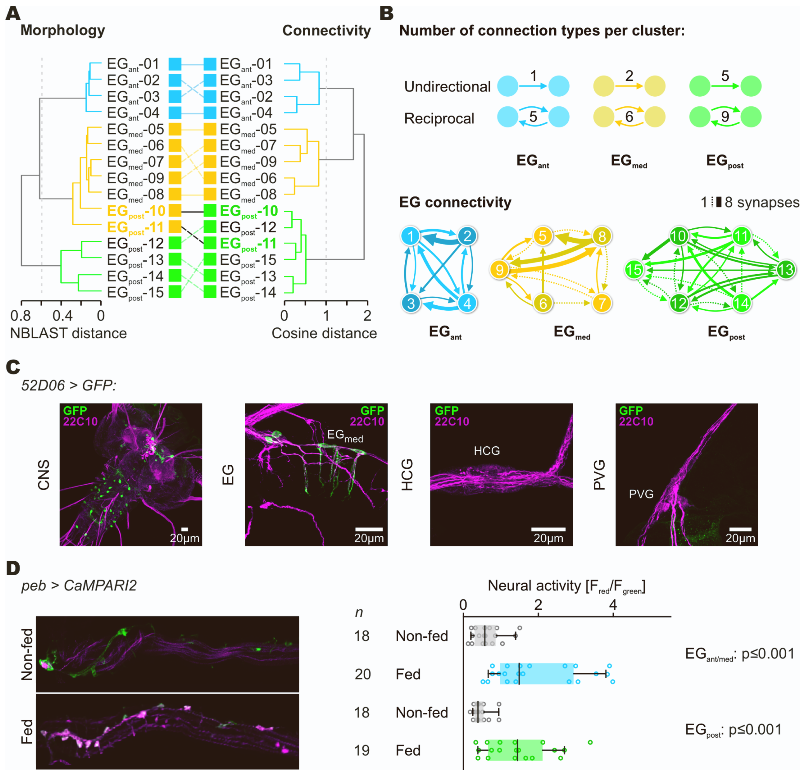

Figure S5. EG neuron connectome and sensory perception upon food intake. Related to Figure 5.

(A) Cluster analysis of EG neurons by neuronal morphology and synaptic connectivity indicates that the EG consists of three entities.

(B) EG connectivity diagram showing the synaptic connections of each individual EG neuron. Line thickness represents the number of synaptic connections. Arrows with dashed lines mark synaptic connections with only one synapse.

(C) Immunohistochemical staining of *52D06 > GFP* showing specific expression in 3-5 cells of EG_{med}. There is no expression in the HCG and the PVG.

(D) Right: representative images of EG show the increased neural activity upon food passage. Left: box plot shows the significantly increased neural activity between non-fed and fed state of $EG_{ant/med}$ and EG_{post}. Performed significance test: Mann-Whitney rank sum test.

Abbr.: $EG_{ant/med/post}$ - esophageal ganglion (anterior, medial, posterior), HCG - hypocerebral ganglion, PVG - proventricular ganglion.

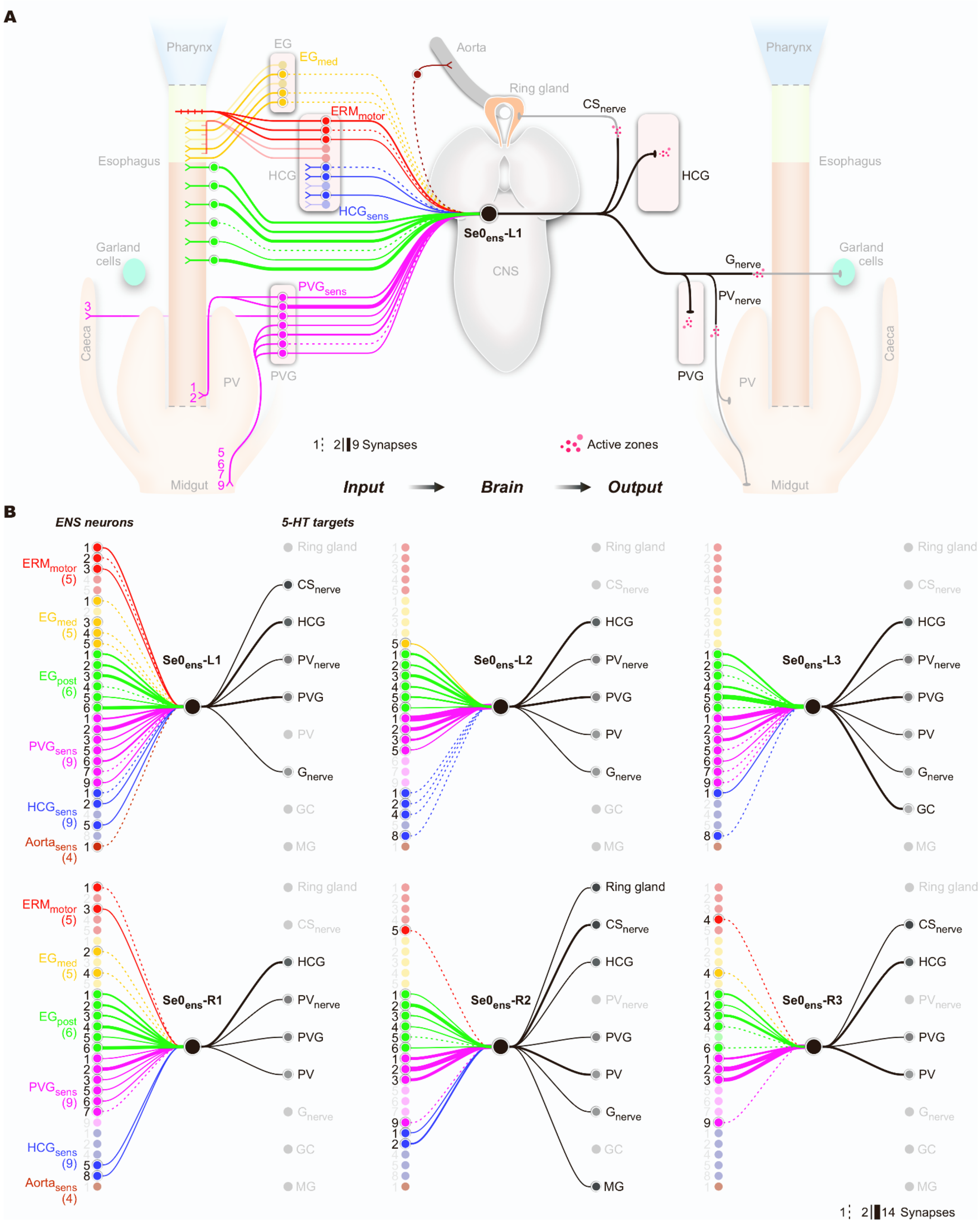

Figure S6. Synaptic inputs and peripheral active zone (outputs) of Se0$_{ens}$ neurons. Related to Figure 6.

(A) Illustration of direct sensory input sources converging on Se0$_{ens}$ neuron combined with diverging active zone output targets relative to ENS and foregut of *Drosophila* larva using the Se0$_{ens}$-L1 neuron as an example.

(B) Flowchart of all six Se0$_{ens}$ neurons showing the direct sensory input sources and active zone output targets in the larval enteric system. Line thickness represents the number of synapses for sensory inputs and active zones for output targets.

Abbr.: Aorta$_{sens}$ - sensory neurons of the aorta, CS$_{nerve}$ – cardio-stomatogastric nerve, EG$_{ant/med/post}$ – esophageal ganglion (anterior, medial, posterior), ERM$_{motor}$ – ERM motor neuron, GC - garland cells, G$_{nerve}$ – garland nerve, HCG$_{sens/VGlut}$ – hypocerebral ganglion (sensory neurons, VGlut-positive neuron), MG - midgut, PV - proventriculus, PV$_{nerve}$ – proventricular nerve, PVG$_{sens}$ – proventricular ganglion (sensory neurons), Se0$_{ens}$ – enteric Se0 neurons.

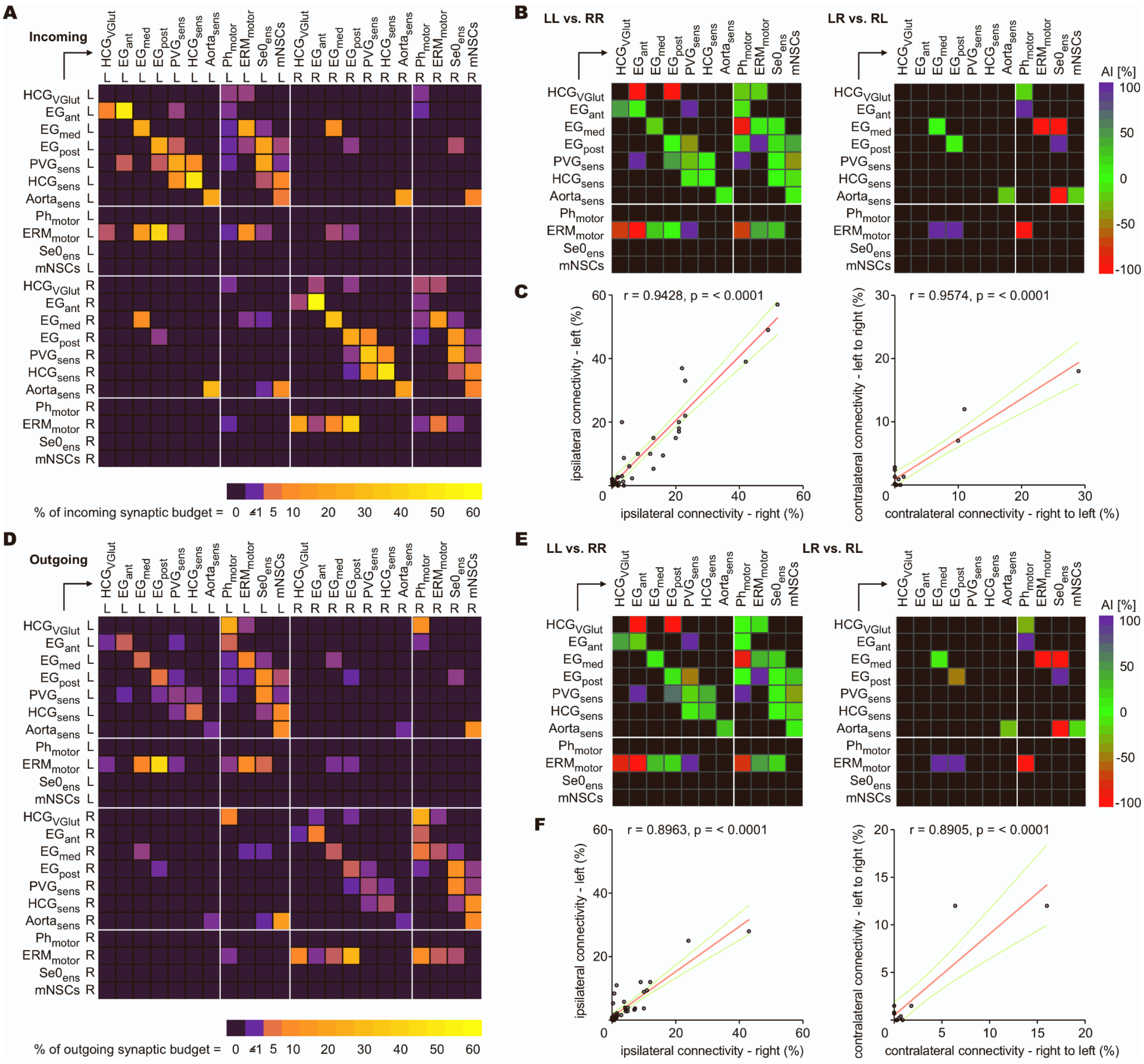

Figure S7. Connectivity symmetry analysis of ENS and Se0 neurons respond to mechano-perception during food intake. Related to Figure 6.

(A) Heat map shows the incoming synaptic budget in percentage between each enteric neuron cluster for the left and right brain hemisphere.

(B) Heat map represents asymmetry index (AI) for the ipsi-(left) and contra-(right)lateral incoming synapses between the clusters of enteric neurons.

(C) Graph shows the correlation between the left/right ipsilateral (left) and left/right contralateral connectivity for incoming synapses of the enteric neurons. Performed correlation test: Spearman's rank correlation test.

(D) Heat map shows the outgoing synaptic budget in percentage between each enteric neuron cluster for the left and right brain hemisphere.

(E) Heat map represents asymmetry index (AI) for the ipsi-(left) and contra-(right)lateral outgoing synapses between the clusters of enteric neurons.

(F) Graph shows the correlation between the left/right ipsilateral (left) and left/right contralateral connectivity for outgoing synapses of the enteric neurons. Performed correlation test: Spearman's rank correlation test.

Abbr.: $Aorta_{sens}$ - sensory neurons of the aorta, $EG_{ant/med/post}$ – esophageal ganglion (anterior, medial, posterior), ERM_{motor} – ERM motor neuron, Ext/ $Ph_{mechano}$ – external/pharyngeal mechanosensory neurons, $HCG_{sens/VGlut}$ – hypocerebral ganglion (sensory neurons, VGlut-positive neuron), mNSCs - medial neurosecretory cells, Ph_{motor} – pharyngeal motor neurons, PVG_{sens} – proventricular ganglion (sensory neurons), $Se0_{ens}$ – enteric Se0 neurons.

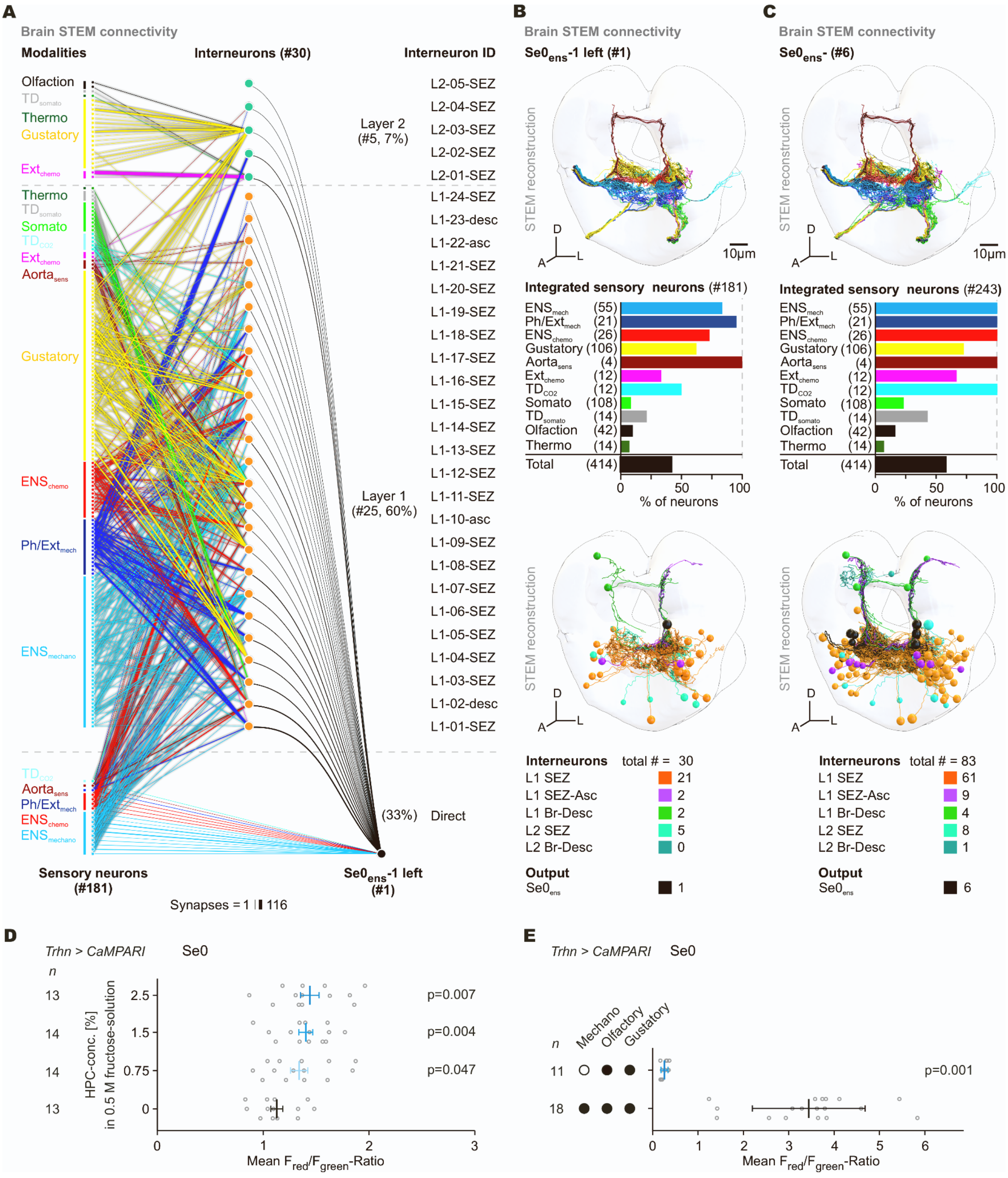

Figure S8. Multimodal sensory integration onto Se0$_{ens}$ through direct and indirect pathways. Related to Figure 6.

(A) Sensory integration onto the single Se0$_{ens}$-L1 neuron at cellular resolution. Via direct and indirect sensory pathways, the Se0$_{ens}$-L1 receives inputs from 181 sensory neurons. The polysynaptic (indirect) pathways comprises two interneuronal layers of 30 interneurons. Data taken from the whole brain STEM volume (Miroschnikow et al., 2018).

(B-C) Comparison of sensory integration by a single Se0$_{ens}$-L1 (B) compared to all Se0$_{ens}$ (C). Top: STEM reconstructions show the all axon of sensory neurons having inputs to Se0$_{ens}$-L1 (B) and Se0$_{ens}$ (C). Bar charts represent the percentage of sensory neurons organized by modality that are directly or indirectly connected to Se0$_{ens}$-L1 (B) and Se0$_{ens}$ (C). Note that the Se0$_{ens}$-L1 maintains nearly the same sensory profile as the entire Se0$_{ens}$. Bottom: STEM reconstructions show all interneurons integrating sensory inputs onto Se0$_{ens}$-L1 (B) and Se0$_{ens}$ (C). Diagram indicates the number of layer 1 and layer 2 interneurons of the indirect sensory integration. Only 30 interneurons integrate sensory information onto Se0$_{ens}$-L1 whereas there are 83 interneurons for the entire Se0$_{ens}$. Data taken from the whole brain STEM volume (Miroschnikow et al., 2018).

(D) Uptake of a 0.5 M fructose solution with different viscosity by addition of hydroxypropyl cellulose (HPC) resulted in increased neural activity of Se0 neurons. Data shows mean ±SE. Performed significance test: student's t-test.

(E) Limiting the yeast experience in larva to the primary external (chemosensory) sense organs for olfaction and gustation by gluing the mouth opening resulted in a decreased neural activity of the Se0 neurons compared to free-behaving larva. Data shows mean ±SE. Performed significance test: Mann-Whitney rank sum test.

Abbr.: Aorta$_{sens}$ - sensory neurons of the aorta, Br-Desc – descending neurons of the brain, ENS$_{chemo/mechano}$ - enteric neuron (chemosensory, mechanosensory), Ext$_{chemo/mechano}$ – external neurons (chemosensory, mechanosensory), Gustatory - gustatory neuron, L1/L2 - layer 1/2, Olfaction - olfactory neuron, Ph$_{mechano}$ - pharyngeal neuron (mechanosensory), Se0$_{ens}$ – enteric Se0 neurons, SEZ - subesophageal zone, SEZ-Asc/Desc – subesophageal zone ascending/descending neurons, Somato - somatosensory neuron, TD$_{CO2/somato}$ - TD neuron (CO_2-sensitive, somatosensory), Thermo - thermosensory neuron.

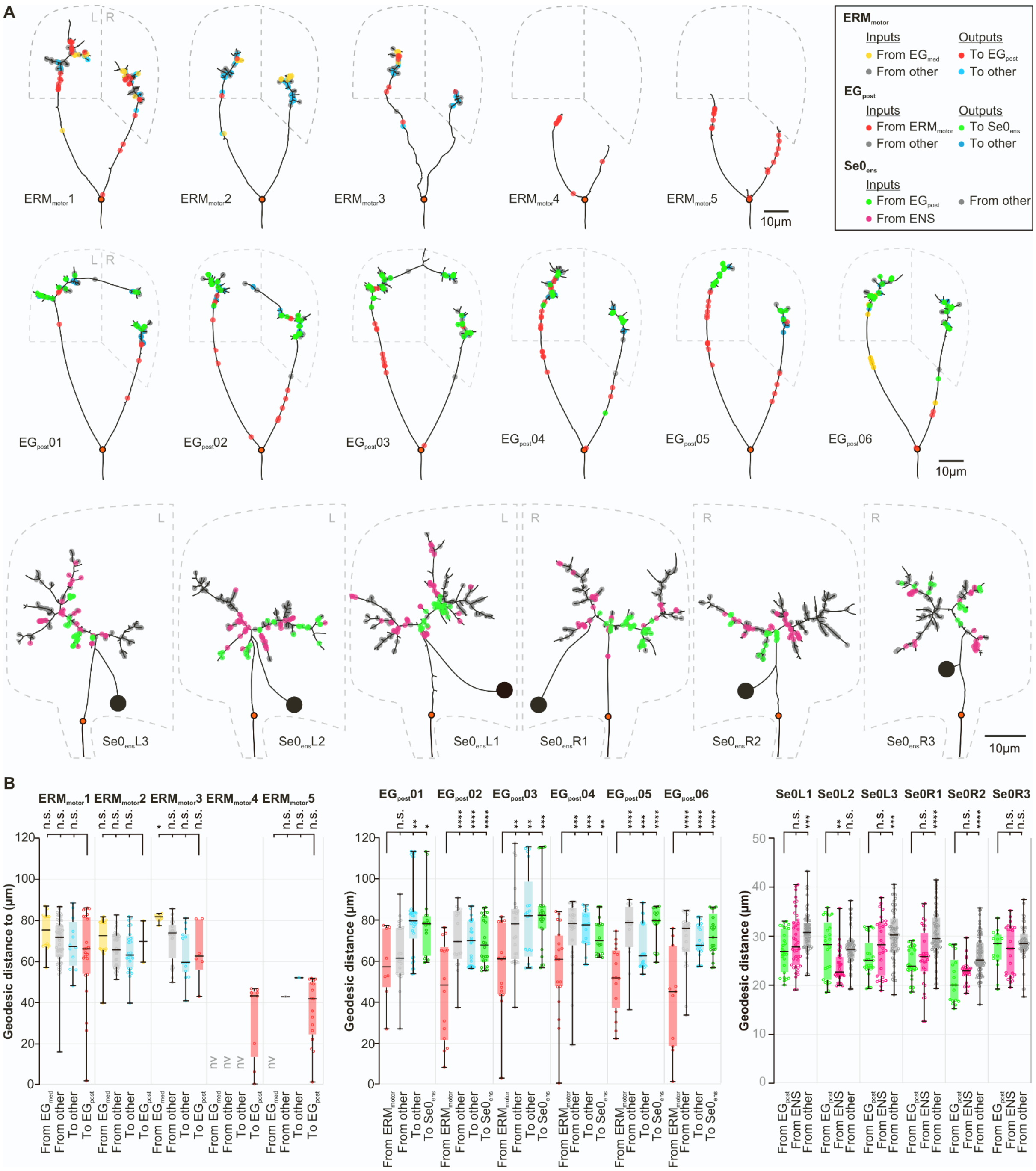

Figure S9. Synapse topology of the swallowing circuit at single cell level. Related to Figure 7.

(A) Two-dimensional dendrograms of all neurons involved the sensorimotor circuit for swallowing.

(B) Spatial synapse analysis for ERM$_{motor}$1-5, EG$_{post}$ 10-15 and Se0$_{ens}$ L/R1-3 at single cell level. Box plots show the geodesic distance of specific pre- and postsynaptic sites for the three neuronal components of the swallowing circuit. Origin for the geodesic distance measurement (orange circle in Figure S7A) is the vagus nerve junction (VNJ) for ERM$_{motor}$/EG$_{post}$ and

nerve entry site for $Se0_{ens}$. Performed statistical test: one-way ANOVA.
Abbr.: ENS - enteric nervous system, ERM_{motor} - Esophageal ring musculature motor neuron, EG_{post} - posterior esophageal ganglion neuron, $Se0_{ens}$ - enteric subesophageal cluster 0 neuron.

	EG_{ant}			EG_{med}			EG_{post}			HCG_{VGlut}			ERM_{motor}			HCG_{sens}			PVG_{mod}			PVG_{sens}		
	mean	std.	n	mean	std.	n	mean	std.	n	mean	std.	n	mean	std.	n	mean	std.	n	mean	std.	n	mean	std.	n
**5-HT1A**	0.077	±0.277	13	1.5	±1.382	12	0.692	±0.855	13	0	±0	18	0	±0	18	5.107	±1.361	14	4.111	±1.875	18	0	±0	18
**5-HT1B**	0.133	±0.352	15	4.067	±0.884	15	0.182	±0.603	11	0	±0	11	0	±0	11	3.818	±0.405	11	5.667	±1.234	15	1.733	±0.458	15
**5-HT2A**	0.0	±0	14	0	±0	14	0	±0	14	0	±0	14	0	±0	14	0	±0	14	0	±0	14	0	±0	14
**5-HT2B**	2.0	±1.08	13	1.154	±1.068	13	0.4	±0.516	10	0	±0	15	0	±0	15	4.615	±0.,87	13	0.04	±0.209	25	3.64	±0.896	25
**5-HT7**	0.667	±0.796	21	0.571	±1.207	21	3.333	±1.623	21	0	±0	20	3	±0.487	20	0	±0	20	3.652	±1.701	23	2.609	±0.902	23

Table S1. Expression-analysis of the enteric nervous system. Related to Figure 2D and S3.
Table shows the number of neurons expressing the serotonin (5-HT) receptor 1A, 1B, 2A, 2B and 7 based on immunohistochemical staining. Listed values are the mean, standard deviation (std.) and number of analyzed enteric nervous systems. Note, that for this analysis data from three different reporter lines were used (GFP, myrGFP and Cam2.1).

	Pharynx (Ph)						Ring gland (RG)									Midgut (MG)														
	PCM expression			ERM expression			PG expression			CC expression			CA expression			MGM_{ant} expression			MGM_{post} expression			EEC_{ant} expression			EEC_{post} expression			EEC expression [%]		
	+	-	n	+	-	n	+	-	n	+	-	n	+	-	n	+	-	n	+	-	n	+	-	n	+	-	n	mean	std.	n
**5-HT1A**	0	13	13	0	13	13	18	0	18	0	18	18	0	18	18	0	13	13	13	0	13	13	0	13	13	0	13	65.071	24.203	152(7)
**5-HT1B**	0	15	15	0	15	15	0	15	15	0	15	15	0	15	15	14	0	14	0	14	14	14	0	14	0	14	14	38.256	22.719	149(7)
**5-HT2A**	0	14	14	0	14	14	0	14	14	0	14	14	0	14	14		8	8	0	8	8	0	8	8	8	0	8	87.039	11.09	51(4)
**5-HT2B**	0	13	13	0	13	13	0	13	13	0	13	13	0	13	13	0	8	8	0	8	8	0	8	8	8	0	8	71.101	16.357	141(4)
**5-HT7**	2	12	14	14	0	21	0	21	21	0	21	21	0	21	21	11	0	11	11	0	11	11	0	11	11	0	11	65.13	20.823	138(4)

Table S2. Expression-analysis of tissues associated with the enteric nervous system. Related to Figure 2D and S3.
Table shows the number of tissues expressing the serotonin (5-HT) receptor 1A, 1B, 2A, 2B and 7 based on immunohistochemical staining. Listed values display the number of 5-HT receptor expressing tissue (+), not 5-HT receptor expressing tissue (-) and total number of analyzed structures (n). Note, that for this analysis data from three different reporter lines were used (GFP, myrGFP and Cam2.1).

Figure	Genotype	Chr.
**2D**<br>(From top to bottom)	*TI{RFP$^{DsRed.3xP3.cUa}$=2A-GAL4}***5-HT1A***/+;*<br>*P{y$^{+t7.7}$ w^{+mC}=10XUAS-mCD8::GFP}attP2/+*	2; 3
	*TI{RFP$^{DsRed.3xP3.cUa}$=2A-GAL4}***5-HT1B***/+;*<br>*P{y$^{+t7.7}$ w^{+mC}=10XUAS-mCD8::GFP}attP2/+*	2; 3
	*TI{RFP$^{DsRed.3xP3.cUa}$=2A-GAL4}***5-HT2A***/*<br>*P{y$^{+t7.7}$ w^{+mC}=10XUAS-mCD8::GFP}attP2*	3
	*TI{RFP$^{DsRed.3xP3.cUa}$=2A-GAL4}***5-HT2B***/*<br>*P{y$^{+t7.7}$ w^{+mC}=10XUAS-mCD8::GFP}attP2*	3
	*TI{RFP$^{DsRed.3xP3.cUa}$=2A-GAL4}***5-HT7***/*<br>*P{y$^{+t7.7}$ w^{+mC}=10XUAS-mCD8::GFP}attP2*	3
**2E**	*Wild type (Oregon-R-C)*	
**2F**<br>(From left to right)	*P{y$^{+t7.7}$ w^{+mC}=GMR29H01-GAL4}attP2/+*	3
	P{y$^{+t7.7}$ w^{+mC}=UAS-TrpA1(B).K}attP16/+	2
	P{y$^{+t7.7}$ w^{+mC}=UAS-TrpA1(B).K}attP16/+;<br>*P{y$^{+t7.7}$ w^{+mC}=GMR29H01-GAL4}attP2/+*	2; 3
**2G**<br>(From left to right)	*Wild type (Oregon-R-C)*	
	P{y$^{+t7.7}$ w^{+mC}=GMR29H01-GAL4}attP2/<br>*PBac{y$^{+mDint2}$ w^{+mC}=UAS-Trhn.miRNA.1}VK00027*	3
**2H**<br>(*$Se0_{ens}$>GCaMP6f*)	*P{y$^{+t7.7}$ w^{+mC}=20XUAS-IVS-GCaMP6f}attP40/+;*<br>*P{y$^{+t7.7}$ w^{+mC}=GMR29H01-GAL4}attP2/+*	2; 3
**3E**<br>(*30F10>nSyb-GFP*)	*P{w^{+mC}=UAS-nSyb.eGFP}2/+;*<br>*P{y$^{+t7.7}$ w^{+mC}=GMR30F10-GAL4}attP2/+*	2; 3
(*30F10>GFP*)	*P{y$^{+t7.7}$ w^{+mC}=GMR30F10-GAL4}attP2/*<br>*P{y$^{+t7.7}$ w^{+mC}=10XUAS-mCD8::GFP}attP2*	3
**3F**<br>(*30F10>GtACR1*)	*P{y$^{+t7.7}$ w^{+mC}=GMR30F10-GAL4}attP2/*<br>*P{y$^{+t7.7}$ w^{+mC}=UAS-GtACR1.d.EYFP}attP2*	3

Figure	Genotype	Chr.
**3G,H**<br>(*30F10>Chrimson*)	P{y$^{+t7.7}$ w^{+mC}=20XUAS-IVS-CsChrimson.mVenus}attP40/+;<br>P{y$^{+t7.7}$ w^{+mC}=GMR30F10-GAL4}attP2/+	2; 3
**4A**<br>(*5-HT7> GFP*)	TI{RFP$^{DsRed.3xP3.cUa}$=2A-GAL4}5-HT7/<br>P{y$^{+t7.7}$ w^{+mC}=10XUAS-mCD8::GFP}attP2	3
(*5-HT7>GFP, VGlut-Gal80*)	P{y$^{+t7.7}$ w^{+mC}=VGlut-GAL80.V}attP40/+;<br>TI{RFP$^{DsRed.3xP3.cUa}$=2A-GAL4}5-HT7/<br>P{y$^{+t7.7}$ w^{+mC}=10XUAS-mCD8::GFP}attP2	2; 3
**4B**<br>(*30F10>GCaMP6f*)	P{y$^{+t7.7}$ w^{+mC}=20XUAS-IVS-GCaMP6f}attP40/+;<br>P{y$^{+t7.7}$ w^{+mC}=GMR30F10-GAL4}attP2/+	2; 3
**4C**<br>(*VGlut>cAMPr*)	P{w^{+mC}=VGlut-GAL4.D}1/+;<br>PBac{y$^{+mDint2}$ w^{+mC}=UAS-cAMPr}VK00037/+	X; 2
**4D**<br>(*30F10>bpac*)	P{w[+mC]=UAS-bPAC.S}2/+;<br>P{y$^{+t7.7}$ w^{+mC}=GMR30F10-GAL4}attP2/+	2; 3
**4E**<br>(*Mef2>GCaMP6s,RFP*)	P{w^{+mC}=UAS-mCD8.mRFP.LG}18a/+; P{w^{+mC}=GAL4-Mef2.R}3/<br>PBac{y$^{+mDint2}$ w^{+mC}=20XUAS-IVS-GCaMP6s}VK00005	2; 3
**4F**<br>(*Mef2>GCaMP6f*)	P{y$^{+t7.7}$ w^{+mC}=20XUAS-IVS-GCaMP6f}attP40/+;<br>P{w^{+mC}=GAL4-Mef2.R}3/+	2; 3
**4G**<br>(From left to right)	Wild type (Oregon-R-C)	
	P{w$^{+mW.hs}$=GawB}VGlut^{OK371} (P{w^{+mC}=UAS-5-HT7.K}) **site of insertion unknown**	2
	P{w$^{+mW.hs}$=GawB}VGlut^{OK371}/+;<br>P{y$^{+t7.7}$ v$^{+t1.8}$=TRiP.JF02576}attP2/+	2; 3
**5B**<br>(*Gr43a>Chrimson*)	TI{GAL4}Gr43a^{GAL4}/<br>P{y$^{+t7.7}$ w^{+mC}=20XUAS-IVS-CsChrimson.mVenus}attP40	2
(*Gr43a>GFP*)	TI{GAL4}Gr43a^{GAL4}/+;<br>P{y$^{+t7.7}$ w^{+mC}=10XUAS-mCD8::GFP}attP2/+	2; 3
**5C**<br>(*52D06>Chrimson*)	P{y$^{+t7.7}$ w^{+mC}=20XUAS-IVS-CsChrimson.mVenus}attP40/+;<br>P{y$^{+t7.7}$ w^{+mC}=GMR52D06-GAL4}attP2/+	2; 3
(*52D06>GFP*)	P{y$^{+t7.7}$ w^{+mC}=GMR52D06-GAL4}attP2/<br>P{y$^{+t7.7}$ w^{+mC}=10XUAS-mCD8::GFP}attP2	3
**5D**<br>(*Piezo>Chrimson*)	P{w^{+mC}=Piezo-GAL4.1.0}IIA/<br>P{y$^{+t7.7}$ w^{+mC}=20XUAS-IVS-CsChrimson.mVenus}attP40	2
(*Piezo>GFP*)	TI{GAL4}Piezo^{KI}/+;<br>P{y$^{+t7.7}$ w^{+mC}=10XUAS-mCD8::GFP}attP2/+	2; 3
**5E**<br>(From left to right)	TI{GAL4}Gr43a^{GAL4}/<br>P{y$^{+t7.7}$ w^{+mC}=20XUAS-IVS-CsChrimson.mVenus}attP40	2
	P{y$^{+t7.7}$ w^{+mC}=20XUAS-IVS-CsChrimson.mVenus}attP40/+;<br>P{y$^{+t7.7}$ w^{+mC}=GMR52D06-GAL4}attP2/+	2; 3
	P{w^{+mC}=Piezo-GAL4.1.0}IIA/<br>P{y$^{+t7.7}$ w^{+mC}=20XUAS-IVS-CsChrimson.mVenus}attP40	2
**5F**<br>(From top to bottom)	Wild type (Oregon-R-C)	
	P{w^{+mC}=Piezo-GAL4.1.0}III/+	3
	P{w^{+mC}=UAS-Piezo.GFP}IIIA/+	3
	PBac{w^{+mC}=RB5.WH5}Piezo^{KO}/ PBac{w^{+mC}=RB5.WH5}Piezo^{KO}	2
	PBac{w^{+mC}=RB5.WH5}Piezo^{KO}/ PBac{w^{+mC}=RB5.WH5}Piezo^{KO};<br>P{w^{+mC}=Piezo-GAL4.1.0}III/+	2; 3
	PBac{w^{+mC}=RB5.WH5}Piezo^{KO}/ PBac{w^{+mC}=RB5.WH5}Piezo^{KO};<br>P{w^{+mC}=UAS-Piezo.GFP}IIIA/+	2; 3
	PBac{w^{+mC}=RB5.WH5}Piezo^{KO}/ PBac{w^{+mC}=RB5.WH5}Piezo^{KO};<br>P{w^{+mC}=Piezo-GAL4.1.0}III/ P{w^{+mC}=UAS-Piezo.GFP}IIIA	2; 3
**5G**<br>(*Piezo>CaMPARI2*)	TI{GAL4}Piezo^{KI}/+;<br>PBac{y$^{+mDint2}$ w^{+mC}=UAS-CaMPARI2}VK00005/+	2; 3
**6E**<br>(*Piezo>TrpA1 Trhn>CaMPARI*)	P{y$^{+t7.7}$ w^{+mC}=UAS-TrpA1(B).K}attP16/+;<br>P{w^{+mC}=Piezo-GAL4.1.0}III/<br>P{y$^{+t7.7}$ w^{+mC}=Trhn-nlsLexA::p65}attP2,<br>PBac{y^{+} w^{+mC}=13XLexAop2-CaMPARI}VK00027	2; 3
**6F**<br>(*Piezo$^{(+/+)}$ Trhn>CaMPARI*)<br>(*Piezo$^{(-/-)}$ Trhn>CaMPARI*)	P{y$^{+t7.7}$ w^{+mC}=Trhn-nlsLexA::p65}attP2,<br>PBac{y^{+} w^{+mC}=13XLexAop2-CaMPARI}VK00027/+	3

Figure	Genotype	Chr.
	PBac{w^{+mC}=RB5.WH5}Piezo^{KO}/ PBac{w^{+mC}=RB5.WH5}Piezo^{KO};<br>*P{y$^{+t7.7}$ w^{+mC}=Trhn-nlsLexA::p65}attP2,*<br>*PBac{y^{+} w^{+mC}=13XLexAop2-CaMPARI}VK00027/+*	2; 3
**S1A**<br>(*5-HT7>GFP*)	*TI{RFP$^{DsRed.3xP3.cUa}$=2A-GAL4}5-HT7/*<br>*P{y$^{+t7.7}$ w^{+mC}=10XUAS-mCD8::GFP}attP2*	3
**S2A**<br>(*Se0$_{ens}$>GFP*)	*P{y$^{+t7.7}$ w^{+mC}=GMR29H01-GAL4}attP2/*<br>*P{y$^{+t7.7}$ w^{+mC}=10XUAS-mCD8::GFP}attP2*	3
(*Se0$_{ph}$>GFP*)	*P{y$^{+t7.7}$ w^{+m}=VT061715-p65.AD}attP40/+;*<br>*P{y$^{+t7.7}$ w^{+m}=VT005008-GAL4.DBD}attP2/*<br>*P{y$^{+t7.7}$ w^{+mC}=10XUAS-mCD8::GFP}attP2*	2; 3
**S2B**<br>(*Trhn>CaMPARI*)	*P{y$^{+t7.7}$ w^{+mC}=UAS-CaMPARI}attP40/+;*<br>*P{w^{+mC}=Trhn-GAL4.long}3/+*	2; 3
**S2C**<br>(*Se0$_{ens}$>CaMPARI*)	*P{y$^{+t7.7}$ w^{+mC}=UAS-CaMPARI}attP40/+;*<br>*P{y$^{+t7.7}$ w^{+mC}=GMR29H01-GAL4}attP2/+*	2; 3
(*Se0$_{ph}$>CaMPARI*)	*P{y$^{+t7.7}$ w^{+m}=VT061715-p65.AD}attP40/*<br>*P{y$^{+t7.7}$ w^{+mC}=UAS-CaMPARI}attP40;*<br>*P{y$^{+t7.7}$ w^{+m}=VT005008-GAL4.DBD}attP2/+*	2; 3
**S3A**<br>(*Se0$_{ens}$>GFP*)	*P{y$^{+t7.7}$ w^{+mC}=GMR29H01-GAL4}attP2/*<br>*P{y$^{+t7.7}$ w^{+mC}=10XUAS-mCD8::GFP}attP2*	3
**S3B**<br>(*Se0$_{ens}$>Brp::GFP,*<br>*CD8::mCherry*)	*P{y$^{+t7.7}$ w^{+mC}=GMR29H01-GAL4}attP2/*<br>*P{w^{+mC}=UAS-brp.S-EGFP}3, P{w^{+mC}=UAS-mCD8.ChRFP}3*	3
**S3E,G**<br>**TableS1, TableS2**<br>(*5-HT1B>GFP*)	*TI{RFP$^{DsRed.3xP3.cUa}$=2A-GAL4}5-HT1B/+;*<br>*P{y$^{+t7.7}$ w^{+mC}=10XUAS-mCD8::GFP}attP2/+*	2; 3
**S3F**<br>**TableS1, TableS2**<br>(*5-HT1A>GFP*)	*TI{RFP$^{DsRed.3xP3.cUa}$=2A-GAL4}5-HT1A/+;*<br>*P{y$^{+t7.7}$ w^{+mC}=10XUAS-mCD8::GFP}attP2/+*	2; 3
**S3H**<br>**TableS1, TableS2**<br>(*5-HT2A>GFP*)	*TI{RFP$^{DsRed.3xP3.cUa}$=2A-GAL4}5-HT2A/*<br>*P{y$^{+t7.7}$ w^{+mC}=10XUAS-mCD8::GFP}attP2*	3
**S3I**<br>**TableS1, TableS2**<br>(*5-HT2B>GFP*)	*TI{RFP$^{DsRed.3xP3.cUa}$=2A-GAL4}5-HT2B/*<br>*P{y$^{+t7.7}$ w^{+mC}=10XUAS-mCD8::GFP}attP2*	3
**S3J**<br>**TableS1, TableS2**<br>(*5-HT7>GFP*)	*TI{RFP$^{DsRed.3xP3.cUa}$=2A-GAL4}5-HT7/*<br>*P{y$^{+t7.7}$ w^{+mC}=10XUAS-mCD8::GFP}attP2*	3
**TableS1, TableS2**<br>(Additionally used reporter lines)	*TI{RFP$^{DsRed.3xP3.cUa}$=2A-GAL4}5-HT1A/+;*<br>*P{y$^{+t7.7}$ w^{+mC}=10XUAS-IVS-myr::GFP}attP2/+*	2; 3
	TI{RFP$^{DsRed.3xP3.cUa}$=2A-GAL4}5-HT1A/<br>*P{w^{+mC}=UAS-Cameleon.2.1}82*	2
	TI{RFP$^{DsRed.3xP3.cUa}$=2A-GAL4}5-HT1B/+;<br>*P{y$^{+t7.7}$ w^{+mC}=10XUAS-IVS-myr::GFP}attP2/+*	2; 3
	TI{RFP$^{DsRed.3xP3.cUa}$=2A-GAL4}5-HT1B/<br>*P{w^{+mC}=UAS-Cameleon.2.1}82*	2
	TI{RFP$^{DsRed.3xP3.cUa}$=2A-GAL4}5-HT2A/<br>*P{y$^{+t7.7}$ w^{+mC}=10XUAS-IVS-myr::GFP}attP2*	3
	P{w^{+mC}=UAS-Cameleon.2.1}82/+;<br>*TI{RFP$^{DsRed.3xP3.cUa}$=2A-GAL4}5-HT2A/+*	2; 3
	TI{RFP$^{DsRed.3xP3.cUa}$=2A-GAL4}5-HT2B/<br>*P{y$^{+t7.7}$ w^{+mC}=10XUAS-IVS-myr::GFP}attP2*	3
	P{w^{+mC}=UAS-Cameleon.2.1}82/+;<br>*TI{RFP$^{DsRed.3xP3.cUa}$=2A-GAL4}5-HT2B/+*	2; 3
	TI{RFP$^{DsRed.3xP3.cUa}$=2A-GAL4}5-HT7/<br>*P{y$^{+t7.7}$ w^{+mC}=10XUAS-IVS-myr::GFP}attP2*	3
	P{w^{+mC}=UAS-Cameleon.2.1}82/+;<br>*TI{RFP$^{DsRed.3xP3.cUa}$=2A-GAL4}5-HT7/+*	2; 3
**S4A**<br>(*30F10>Cam2.1*)	*P{w[+mC]=UAS-Cameleon.2.1}82/+;*<br>*P{y$^{+t7.7}$ w^{+mC}=GMR30F10-GAL4}attP2/+*	2; 3
**S4B**<br>(*OK371>Epac-camps*)	*P{w$^{+mW.hs}$=GawB}VGlut^{OK371}/*<br>*P{w^{+mC}=UAS-Epac1-camps}50A*	2

Figure	Genotype	Chr.
**S5C** (*52D06>GFP*)	*P{y$^{+t7.7}$ w^{+mC}=GMR52D06-GAL4}attP2/ P{y$^{+t7.7}$ w^{+mC}=10XUAS-mCD8::GFP}attP2*	3
**S5D** (*peb>CaMPARI2*)	*P{w^{+m*}=GAL4}peb/+; PBac{y$^{+mDint2}$ w^{+mC}=UAS-CaMPARI2}VK00005/+*	X; 3
**S8D,E** (*Trhn>CaMPARI*)	*P{y$^{+t7.7}$ w^{+mC}=UAS-CaMPARI}attP40/+; P{w^{+mC}=Trhn-GAL4.long}3/+*	2; 3

Table S3. Genotypes of experimental flies used in each figure and supplementary figure. Related to Figures 2-6; S1-S5 and S8; Table S1,S2.
